# Acetylation of Beclin 1 inhibits autophagosome maturation and promotes tumour growth

**DOI:** 10.1038/ncomms8215

**Published:** 2015-05-26

**Authors:** Ting Sun, Xuan Li, Peng Zhang, Wen-Dan Chen, Hai-liang Zhang, Dan-Dan Li, Rong Deng, Xiao-Jun Qian, Lin Jiao, Jiao Ji, Yun-Tian Li, Rui-Yan Wu, Yan Yu, Gong-Kan Feng, Xiao-Feng Zhu

**Affiliations:** 1 State Key Laboratory of Oncology in South China, Collaborative Innovation Center for Cancer Medicine, Cancer Center, Sun Yat-sen University, Guangzhou 510060, China; 2 Department of Clinical Laboratory, The First Affiliated Hospital of Zhengzhou University, Zhengzhou 450002, China

## Abstract

Beclin 1, a protein essential for autophagy, regulates autophagy by interacting with Vps34 and other cofactors to form the Beclin 1 complex. Modifications of Beclin 1 may lead to the induction, inhibition or fine-tuning of the autophagic response under a variety of conditions. Here we show that Beclin 1 is acetylated by p300 and deacetylated by SIRT1 at lysine residues 430 and 437. In addition, the phosphorylation of Beclin 1 at S409 by CK1 is required for the subsequent p300 binding and Beclin 1 acetylation. Beclin 1 acetylation inhibits autophagosome maturation and endocytic trafficking by promoting the recruitment of Rubicon. In tumour xenografts, the expression of 2KR mutant Beclin 1 (substitution of K430 and K437 to arginines) leads to enhanced autophagosome maturation and tumour growth suppression. Therefore, our study identifies an acetylation-dependent regulatory mechanism governing Beclin 1 function in autophagosome maturation and tumour growth.

## Abstract (web summary)

Beclin 1 is an essential autophagy effector, necessary to form the autophagosome. Here Sun *et al.* show that Beclin 1 acetylation regulated by p300 and SIRT1 inhibits autophagosome maturation, and mutation of the acetylation sites leads to tumour growth suppression *in vivo*.

## Graphical abstract

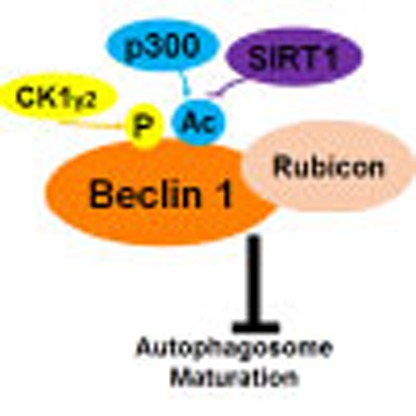

Autophagy is a lysosome-dependent cellular degradation process that functions in nutrient recycling, energy generation and the clearance of damaged proteins and organelles^1^. Cytoplasmic materials targeted for autophagic destruction are sequestered into newly emerging double-membrane vesicles called autophagosomes, and delivered for lysosomal degradation^2^. Autophagy contributes to survival during starvation and other forms of cellular stress; it also functions in differentiation and development, anti-aging, innate and adaptive immunity, and tumour suppression^2^,^3^,^4^,^5^.

Beclin 1, the mammalian orthologue of yeast Atg6/Vps30, is an essential autophagy effector and has an important role in development, tumorigenesis and neurodegeneration^6^. It is established that the overall activity of Vps34 is positively regulated by Beclin 1. Vps34 is the Class III phosphoinositide 3-kinase that phosphorylates phosphatidylinositol to generate phosphatidylinositol 3-phosphate, which is essential for both intracellular trafficking and autophagosome formation^7^. Beclin 1 can regulate autophagy by combining with Vps34, and other positive and negative cofactors such as Atg14L/Barkor, UVRAG, Rubicon, Bcl-2 and Bcl-XL to form the Beclin 1 complex^6^,^8^,^9^,^10^,^11^,^12^,^13^,^14^. Although the Beclin 1 complex has been studied extensively, little is known about the molecular mechanism underlying the conversion of autophagosome to degradative autolysosome.

Protein acetylation has been reported to play a role in autophagy regulation^15^,^16^. There is now accumulating evidence for Atg protein regulation by acetylation. However, it remains unclear whether Beclin 1 is regulated by acetylation.

Here we identify acetylation as a novel post-translational modification of Beclin 1. We demonstrate that Beclin 1 is acetylated and deacetylated by p300 and SIRT1, respectively, at K430 and K437. The CK1-mediated phosphorylation of Beclin 1 at S409 enhances the subsequent binding of Beclin 1 to p300 and the acetylation of Beclin 1. Beclin 1 acetylation inhibits autophagosome maturation promoting the recruitment of Rubicon. In tumour xenografts, the acetylation-deficient mutant Beclin 1–2KR inhibits cell proliferation and tumour growth. Therefore, we identify the molecular mechanism by which acetylation regulates Beclin 1 function in autophagosome maturation and tumour growth.

## Results

### Beclin 1 is acetylated at lysine 430 and 437

**Figure 1:**
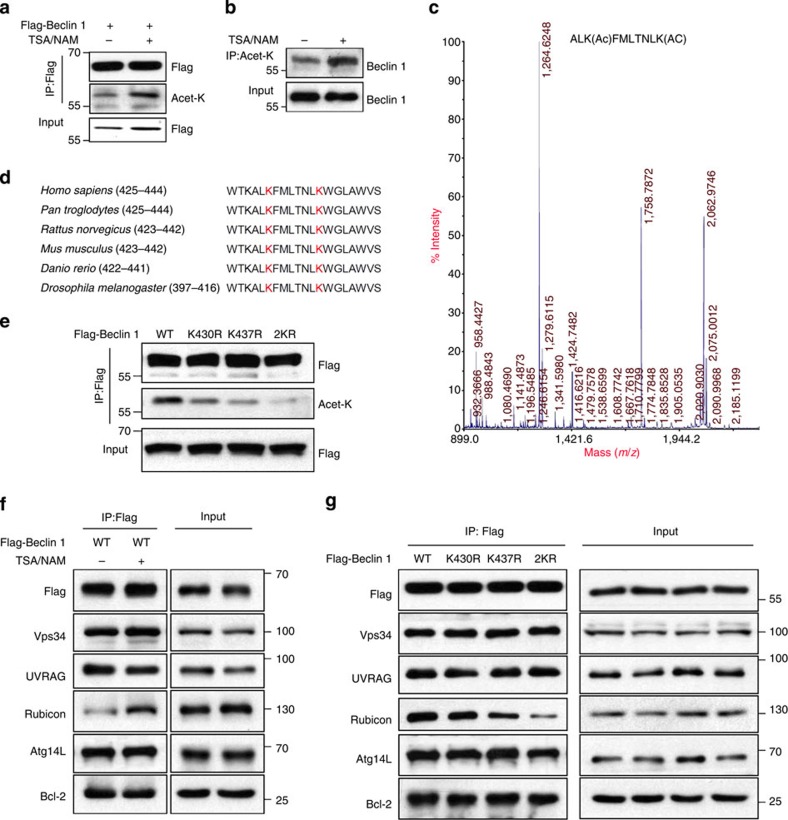
Beclin 1 is acetylated at lysines 430 and 437. (**a**) Exogenous Beclin 1 is acetylated. Acetylation of immunoprecipitated Flag-tagged Beclin 1 from HEK293T cells treated with or without HDAC inhibitors TSA (1 μM) and NAM (5 mM) simultaneously for 6 h. (**b**) Endogenous Beclin 1 is acetylated. Acetylated proteins were immunoprecipitated with the antibody to acetylated lysine from HEK293T cells after TSA and NAM treatments as indicated. Acetylation of endogenous Beclin 1 protein was analysed with western bolt analysis. (**c**) Identification of Beclin 1 K430 and K437 acetylation using mass spectrometry analysis. Flag-tagged Beclin 1 was transfected into HEK293T cells. At 24 h post transfection, TSA (1 μM) and NAM (5 mM) were added for another 6 h. Beclin 1 was purified by immunoprecipitation with an anti-Flag antibody and then analysed using mass spectrometry. (**d**) Alignment of the protein sequences of Beclin 1 homologues in various species. The red indicates the identified acetylated lysine residues of Beclin 1. (**e**) Mutations of K430 and K437 decrease Beclin 1 acetylation. Acetylation of ectopically expressed WT, K430R, K437R and Beclin 1–2KR was analysed. (**f**) TSA and NAM increase the binding of Beclin 1 to Rubicon. Immunoprecipitation of indicated Beclin 1-binding partners with ectopically expressed Flag-Beclin 1 in HEK293T cells treated with TSA and NAM. (**g**) Mutations of K430 and K437 decrease the binding of Beclin 1 and Rubicon. Immunoprecipitation of indicated proteins with Beclin 1 in HEK293T cells transfected with indicated Beclin 1 constructs.

Mass spectrometry-based proteomic analyses have recently identified a large number of potentially acetylated proteins^17^. To confirm the acetylation status of Beclin 1, human embryonic kidney (HEK) 293T cells were transiently transfected with Flag-tagged Beclin 1, and the acetylation level of ectopically expressed Beclin 1 was detected by using an anti-acetylated lysine antibody. We found that the level of acetylated Beclin 1 was significantly increased after treating cells with nicotinamide (NAM), which is an inhibitor of the SIRT family deacetylases, and simultaneously with trichostatin A (TSA), which is an inhibitor of histone deacetylase (HDAC) classes I, II and IV (Fig. 1a). Similar experiments with endogenous Beclin 1 also showed that TSA and NAM treatments enhanced Beclin 1 acetylation (Fig. 1b).

To identify the acetylation sites on Beclin 1, ectopically expressed Beclin 1 was purified and then analysed using mass spectrometry. From three independent experiments, we repeatedly identified a dual acetyl-lysine-containing peptide (ALKAcFMLTNLKAc) that was mapped to a region containing K430 and K437 on human Beclin 1 (Fig. 1c). A protein sequence alignment of Beclin 1 homologues from different species showed that K430 and K437 are evolutionarily conserved from *Caenorhabditis elegans* to mammals (Fig. 1d). When we mutated each or both lysines (K) to arginines (R), each single mutation resulted in a weak reduction in Beclin 1 acetylation, whereas the double mutation (2KR) resulted in a significant decrease in Beclin 1 acetylation (Fig. 1e), indicating that Beclin 1 can be acetylated at the sites of Lys430 and Lys437.

### Acetylation alters the Beclin 1 complex

Given that the overall activity of the Vps34 complex is regulated by Beclin 1 and a set of interacting components, we hypothesized that the acetylation of Beclin 1 might alter the Beclin 1 complex. To test this hypothesis, we examined the interaction between Beclin 1 and its binding partners after treating HEK293T cells with TSA and NAM. These deacetylase inhibitors did not affect the interaction of Beclin 1 with Atg14L, Vps34, UVRAG and Bcl-2; however, they increased the co-immunoprecipitation of Beclin 1 and Rubicon (Fig. 1f). When K430 or K437 was mutated to arginine, we found that each single mutation weakens the binding of Beclin 1 to Rubicon. However, when both residues were mutated, the interaction of Beclin 1–2KR with Rubicon was almost completely abolished. In contrast, these mutations did not affect the interaction of Beclin 1 with the other binding partners (Fig. 1g). We also confirmed the results in human breast adenocarcinoma MCF7 cells, which was consistent with those observed in 293T cells (Supplementary Fig. 1a). Meanwhile, we constructed two plasmids with KL and KQ mutant to mimic the lysine acetylation as previously reported. As shown in the Supplementary Fig. 1b–d, the KQ mutantion had no obvious effect on the Beclin 1/Rubicon interaction, and KL mutation slightly decreased the interaction compared with Beclin 1 Wild Type (WT). Since they both could not enhance the binding of Beclin 1 with Rubicon, it suggested that neither KQ nor KL mutation could mimic the effect of Beclin 1 acetylation. Some studies argued that the effects of *in vivo* acetylation might be overestimated when the KQ mutant was used as a mimic of the acetylated lysine, and there existed many differences between glutamine and acetyl lysine, such as the distribution of atoms and charges, which could further affect the residue interaction^18^. Together, these data indicate that acetylation in Lys430 and Lys437 regulates the Beclin 1 complex by promoting the binding of Rubicon to Beclin 1.

### p300 acetylates Beclin 1

**Figure 2:**
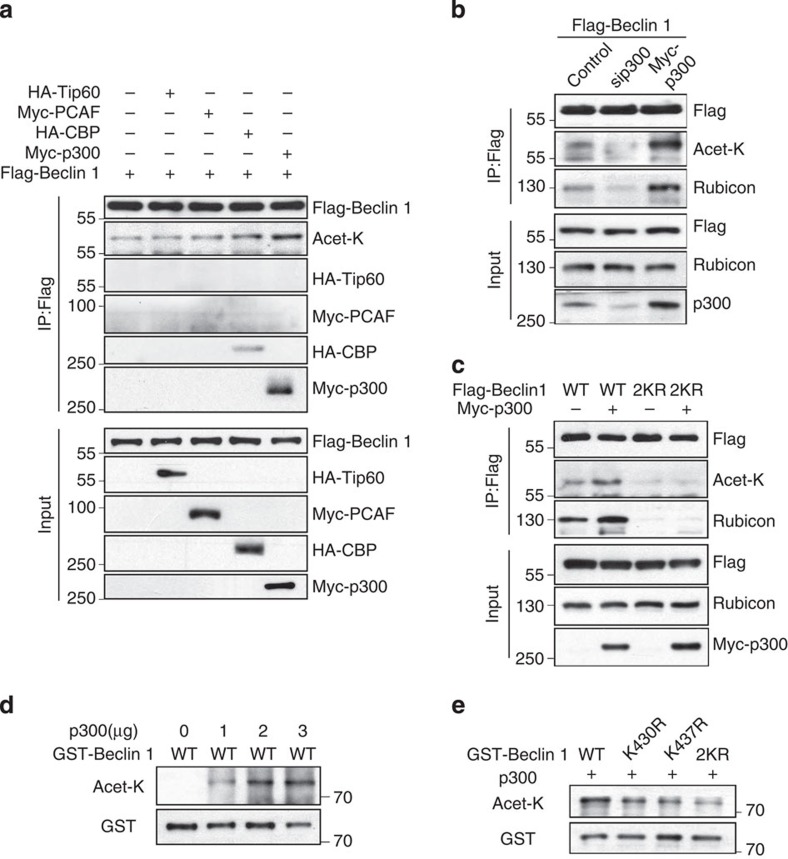
p300 is the acetyltransferase of Beclin 1. (**a**) Overexpression of p300, but not other acetyltransferases (HATs), could significantly bind with Beclin 1 and increase Beclin 1 acetylation. HA-tagged CBP or Tip60 or Myc-tagged p300 or p300/CBP-associated factor (PCAF) was co-transfected individually with Flag-Beclin 1 into HEK293T cells. (**b**) Knockdown or overexpression of p300 alters Beclin 1 acetylation and Beclin 1–Rubicon interaction. Flag-Beclin 1 was co-transfected with si*p300*, Myc-p300 or vector into HEK293T cells, Beclin 1 acetylation and the interaction between Beclin 1 and Rubicon was determined with IP and western blot analyses. (**c**) Deacetylation mimic (2KR) counteracts p300 acetylation of Beclin 1 and diminishes the binding of Beclin 1 with Rubicon. Flag-tagged Beclin 1 (WT, 2KR) was co-transfected with Myc-p300 or vector into HEK293T cells. (**d**) Beclin 1 is acetylated by p300 *in vitro*. Different doses of recombinant human p300 were incubated with bacterially expressed GST–Beclin 1 in the presence of 100 μM AcCoA for 1 h at 30 °C. (**e**) Sequential mutation of two acetylable lysine residues (K430 and K437) to arginine progressively diminished the acetylation of GST–Beclin 1 by p300 *in vitro*.

To identify the acetyltransferase responsible for Beclin 1 acetylation at K430 and K437, we transfected four acetyltransferases, including Tip60 (TAT-interacting proteins 60, also known as KAT5), PCAF (p300/CBP-associated factor, also known as KAT2B), CBP (cAMP response element-binding protein) and p300 (E1A-binding protein, 300 kDa), individually into HEK293T cells. We found that the ectopic expression of p300 could bind with Beclin 1 and significantly increase Beclin 1 acetylation, whereas the other acetyltransferases had little effect (Fig. 2a, Supplementary Fig. 2). Notably, p300 knockdown reduced Beclin 1 acetylation, while p300 overexpression enhanced Beclin 1 acetylation (Fig. 2b). Moreover, the overexpression of p300 increased the interaction of Rubicon with Beclin 1 WT but not with Beclin 1–2KR mutant (Fig. 2c). Furthermore, an *in vitro* acetylation assay revealed that p300 acetylated the glutathione S-transferase (GST)-tagged Beclin 1 fusion protein dose-dependently (Fig. 2d) and that the level of acetylation was reduced with the single (K430R or K437R) and double (2KR) mutants (Fig. 2e). Therefore, our results indicate that mutations of these two residues reduced the p300-mediated acetylation of Beclin 1.

### SIRT1 deacetylates Beclin 1

**Figure 3:**
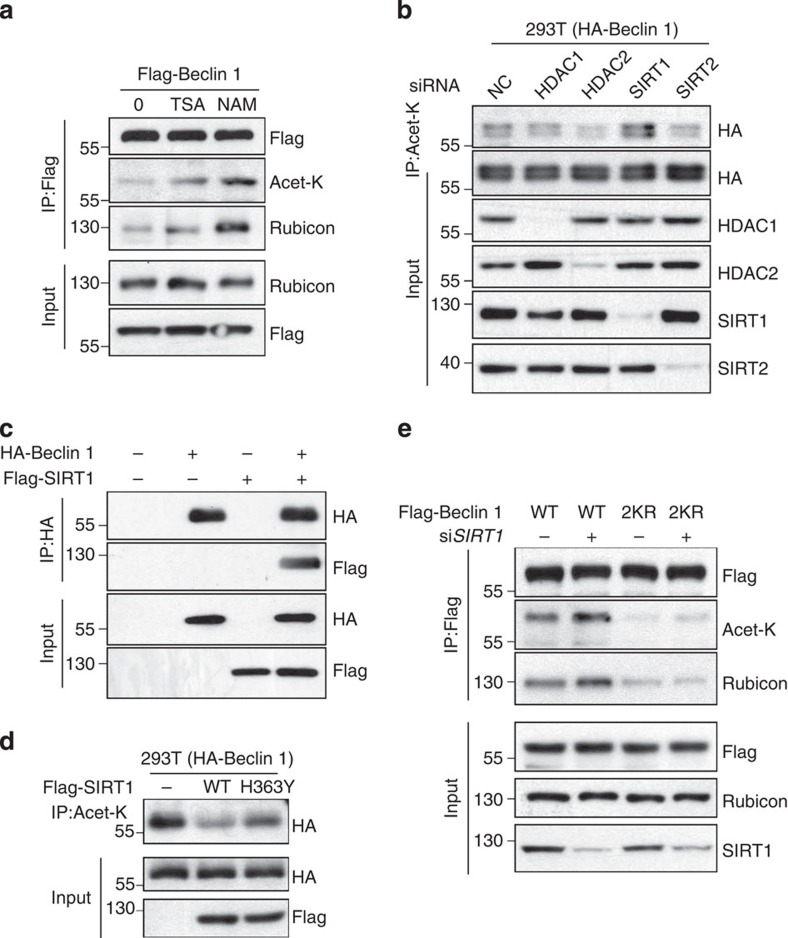
SIRT1 decreases Beclin 1 acetylation and the interaction of Beclin 1 and Rubicon. (**a**) NAM, not TSA, increases Beclin 1 acetylation. Flag-tagged Beclin 1 was transfected into HEK293T cells with or without sirtuin deacetylase inhibitor NAM and HDAC inhibitor TSA. Acetylation of Beclin 1 was measured with IP western blot analyses. (**b**) SIRT1 knockdown increases Beclin 1 acetylation. HEK293T cells stably expressing HA-Beclin 1 were transfected with siRNA targeting HDAC1, HDAC2, SIRT1 and SIRT2 or negative control. Beclin 1 acetylation was measured by immunoprecipitation using an anti-acetylated lysine antibody. (**c**) Association of Beclin 1 with SIRT1. HA-tagged Beclin 1 and Flag-tagged p300 were transfected into HEK293T cells individually or together. The interaction between Beclin 1 and SIRT1 was determined with IP and western blot analyses. (**d**) Catalytic activity of SIRT1 is required for deacetylation of Beclin 1. HEK293T cells stably expressing HA-Beclin 1 were transfected into Flag-tagged SIRT1 WT or catalytically inactive mutant H363Y. Beclin 1 acetylation was measured by immunoprecipitation using an anti-acetylated lysine antibody. (**e**) Beclin 1–2KR-mutant counteracts si*SIRT1* acetylation of Beclin 1 and diminishes the Beclin 1–Rubicon interaction. Flag-tagged Beclin 1 (WT, 2KR) was co-transfected with si*SIRT1* or negative control into HEK293T cells.

To identify the deacetylase that targets K430 and K437 on Beclin 1, we used the deacetylase inhibitors TSA and NAM to determine whether HDACs or SIRTs are involved in Beclin 1 deacetylation. Treatment of cells with the SIRT1 inhibitor NAM had a greater effect on Beclin 1 acetylation than the HDAC inhibitor TSA (Fig. 3a), indicating that the SIRT family may be preferentially involved in Beclin 1 deacetylation. We confirmed this result using small interfering RNA (siRNA) to knockdown HDAC1, HDAC2, SIRT1 or SIRT2, and found that only the si*SIRT1* increased Beclin 1 acetylation (Fig. 3b). We further confirmed this result using two effective siRNAs targeting different sequences of *SIRT1* (Supplementary Fig. 3a). Beclin 1 was readily co-precipitated with SIRT1 when both proteins were overexpressed in HEK293T cells (Fig. 3c). Furthermore, the expression of wild type, but not the catalytically inactive SIRT1 mutant (H363Y), led to a decrease in Beclin 1 acetylation (Fig. 3d). Importantly, the knockdown of SIRT1 also resulted in the enhanced interaction of Rubicon with wild-type but not with the 2KR mutant Beclin 1 (Fig. 3e). Moreover, we confirmed our finding in MCF7 cells, and the result was consistent with those observed in 293T cell (Supplementary Fig. 3b). Furthermore, an *in vitro* deacetylation assay revealed that SIRT1 could deacetylate the immunoprecipitated WT Beclin 1, which could be restored on the NAM pretreatment (Supplementary Fig. 3c). Together, these results demonstrate that SIRT1 is the primary deacetylase for Beclin 1 and that Beclin 1 deacetylation weakens its interaction with Rubicon.

### CK1 promotes acetylation of Beclin 1 by p300

**Figure 4:**
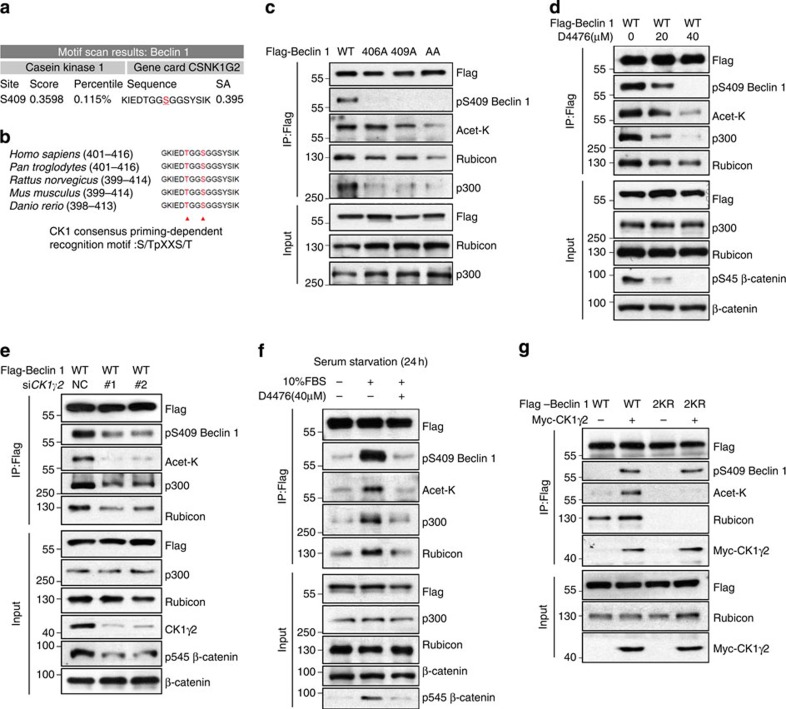
CK1-mediated phosphorylation of Beclin 1 is essential for Beclin 1 acetylation. (**a**) Motif scan result of Beclin 1 by Scansite. Choosing the stringency level is high. (**b**) Cluster alignment of CK1 consensus phosphorylation motif on Beclin 1 proteins. Arrowheads point to T406 and S409 of human Beclin 1. (**c**) Mutations of T406 and S409 decrease Beclin 1 phosphorylation and acetylation. HEK293T cells were transfected with indicated Beclin 1 constructs. Beclin 1 phosphorylation, acetylation and the Beclin 1–Rubicon interaction were detected by IP as indicated. (**d**) Inhibition of CK1 with D4476 decreases Beclin 1 acetylation. HEK293T cells transfected with Flag-Beclin 1 were treated with indicated concentration of D4476 for 4 h. Beclin 1 acetylation and the interaction between Beclin 1 and p300 or Rubicon was determined with IP and western blot analyses. (**e**) CK1γ2 knockdown decreases the binding of p300 to Beclin 1 and Beclin 1 acetylation. Flag-Beclin 1 was co-transfected with siRNA into HEK293T cells. Beclin 1 acetylation and the interaction between Beclin 1 and p300 or Rubicon was determined with IP and western blot analyses. (**f**) Serum restoration increases Beclin 1 acetylation by activating CK1. HEK293T cells transfected with Flag-Beclin 1 were serum-starved for 24 h, and then serum was restored with or without D4476 pretreatment (40 μM, 2 h). (**g**) Overexpression of CK1γ2 increases WT Beclin 1 acetylation but not 2KR mutant. Flag-tagged Beclin 1 (WT, 2KR) was co-transfected with Myc-CK1γ2 or vector into HEK293T cells.

Scansite is a short linear motif-based scanning approach for identifying sequence motifs that are likely to be phosphorylated by specific protein kinases or bind to specific protein domains, such as 14-3-3, SH2 and SH3 domains^19^. We predicted the possible phosphorylation sites for particular protein kinases in Beclin 1 using Scansite and found that Beclin 1 is likely phosphorylated at Ser409 by CK1γ2 (Casein kinase 1 gamma 2, CSNK1G2; Fig. 4a). This result was confirmed using mass spectrometry, which also identified T406 as another potential phosphorylation site (Supplementary Table 1). Phosphorylation by the CK1 kinase family typically requires priming phosphorylation at the −3 position^20^. The consensus priming-dependent recognition motif is S/Tp-X-X-S/T, where S/Tp refers to a phosphoserine or phosphothreonine and the underlined residues refer to the target site (Fig. 4b). Thus, it is likely that T406 is a priming phosphorylation site for S409 phosphorylation catalysed by CK1.

To confirm Beclin 1 phosphorylation by CK1γ2, we generated a phospho-specific antibody against Beclin 1 S409. In an *in vitro* assay, we found that the GST-tagged Beclin 1 fusion protein could be phosphorylated by the active CK1γ2 kinase at the Ser409 residue (Supplementary Fig. 4a). Using site-directed mutagenesis, we mutated the potential phosphorylation sites on Beclin 1 and found that the pS409-Beclin 1 antibody only recognized Beclin 1 WT, but not the S409A or the T406A/S409A (AA) mutant Beclin 1, when Beclin 1 and CK1γ2 were ectopically co-expressed (Fig. 4c). Interestingly, very little S409 phosphorylation was detected when the T406A Beclin 1 mutant was expressed (Fig. 4c). These data indicate that S409 is the CK1γ2 target phosphorylation site and that T406 may be the priming phosphorylation site. Furthermore, while the T406A and S409A mutants showed decreased phosphorylation by CK1γ2, we also found that both the level of Beclin 1 acetylation and the interaction between Beclin 1 and p300 were decreased in mutant cells (Fig. 4c), whereas the phosphomimetic mutants could enhance the acetylation and the Beclin 1–p300 interaction (Supplementary Fig. 4b), suggesting that the phosphorylation of Beclin 1 by CK1γ2 may promote Beclin 1 acetylation by enhancing its binding with p300, and subsequently the formation of Beclin 1–Rubicon complex. To further validate our finding, we blocked CK1 activity with D4476, which is a cell-permeable inhibitor of the CK1 kinase family. We also examined the Ser45 phosphorylation of β-catenin (reported as a substrate of CK1) to further confirm the inhibition of CK1γ2. After D4476 treatment, Beclin 1 acetylation and interaction with Rubicon were decreased, likely due to attenuated binding of p300 to Beclin 1(Fig. 4d and Supplementary Fig. 4c). Similar effects were observed when CK1γ2 was knocked down (Fig. 4e). CK1 can be activated by serum restoration after cells are serum-starved for 24 h. Using this approach, we found that serum stimulation after starvation resulted in enhanced Beclin 1–Rubicon interaction and increased Beclin 1 acetylation in HEK293T cells, which corresponded to enhanced interaction between Beclin 1 and p300, and these effects can be counteracted by D4476 treatment (Fig. 4f). Meanwhile, we confirmed the level of Beclin 1 phosphorylation at S409, which was decreased after CK1γ2 knockdown or treatment with CK1 inhibitor D4476 (Fig. 4d,e and Supplementary Fig. 4c) and increased on serum restoration (Fig. 4f). Moreover, the overexpression of CK1γ2 increased the interaction of Rubicon with wild-type Beclin 1 but not with 2KR mutant Beclin 1 (Fig. 4g). These results further indicate that the CK1γ2-mediated phosphorylation of Beclin 1 may enhance the subsequent interaction between Beclin 1 and p300, thus promoting Beclin 1 acetylation.

### Acetylation of Beclin 1 inhibits autophagosome maturation

**Figure 5:**
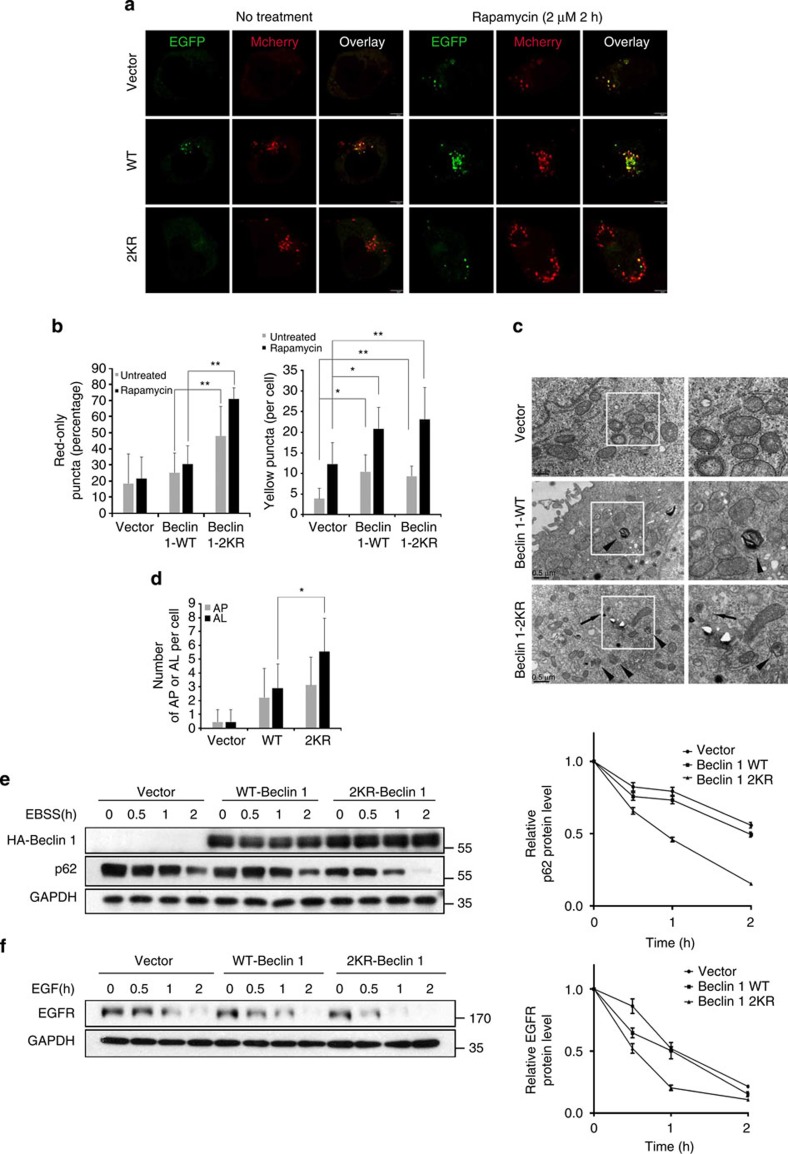
Autophagosome maturation and endocytic trafficking in Beclin 1–2KR-expressing cells. (**a**) Beclin 1–2KR mutant promotes the maturation of autophagosomes. 293T cells stably expressing Vector control, Beclin 1 WT or 2KR were transfected with mCherry-EGFP-LC3 in the absence or presence of rapamycin (2 μM). Scale bars, 5 μm. (**b**) Quantitation of mCherry-LC3-only puncta and the mCherry-EGFP overlay puncta in 293T cell lines treated as in **a**. Bars represent mean±s.d. of 50 cells; five independent experiments, *P<0.05, **P<0.01 (Student's *t*-test). (**c**) Electron microscopy analysis of autophagosomes and autolysosomes. MCF7 cells stably expressing Beclin 1 (WT or 2KR) or Vector grown in normal medium was subjected to electron microscopy analysis. The arrows indicate autophagosomes (AP) and the arrowheads indicate autolysosomes (AL). Scale bar, 0.5 μm. (**d**) Quantitation of AP and AL in MCF7 cell lines as in **c**. Bar are mean ±s.d. of 20 cells; three independent experiments, *P<0.05 (Student's *t*-test). (**e**) The effect of Beclin 1–2KR mutant on p62 turnover. MCF7 cells stably expressing vector control, Beclin 1 WT or 2KR were treated with EBSS for the indicated times. The band intensity was measured in three independent experiments and the mean±s.d. are shown (down panel). (**f**) EGFR degradation. MCF7 cells stably expressing vector control, Beclin 1 WT or 2KR were serum-starved for 24 h, and then treated with EGF (200 ng ml^−1^) for the indicated times. The band intensity was measured in three independent experiments and the mean±s.d. are shown (down panel).

Rubicon is a negative regulator for autophagosome and endosome maturation^6^,^14^. Given our observation that Beclin 1 acetylation promoted Beclin 1–Rubicon interaction, we hypothesized that it might also inhibit autophagosome maturation and endocytosis. To test this hypothesis, we stably transfected Vector control, wild-type Beclin 1 or the 2KR mutant into 293T cells. We then assessed the effect of Beclin 1 acetylation on autophagosome maturation using the mCherry-EGFP (enhanced green fluorescent protein)-LC3 tandem vector, which was used to distinguish the autophagosomes (EGFP-positive) and autolysosomes (mCherry only that due to quenching of EGFP in the acidic environment of lysosome)^21^. The percentage of red-only puncta was significantly higher in cells expressing Beclin 1–2KR than in the ones expressing Beclin 1 WT, and this tendency was further enhanced when treated with the mammalian target of rapamycin (mTOR) inhibitor rapamycin (Fig. 5a,b). At the final step of maturation, autophagosome fuses with lysosomes. Therefore, we also examined the colocalization efficiency of GFP–LC3-labelled autophagosomes and LAMP2-stained late endosomes/lysosomes. The percentage of GFP-LC3+/LAMP2+ staining was significantly higher in HeLa cells expressing Beclin 1–2KR than that in HeLa cells expressing Beclin 1 WT, and the colocalization was further enhanced when treated with the mTOR inhibitor rapamycin (Supplementary Fig. 5a). Nonetheless, treatment with bafilomycin A1, a specific inhibitor of vacuolar H+-ATPase, significantly blocked the autophagosome maturation in Beclin 1–2KR-expressing cells (Supplementary Fig. 5a). Human breast adenocarcinoma MCF7 cells express low levels of endogenous Beclin 1 and we stably transfected Vector control, Beclin 1 WT or the 2KR mutant into MCF7 cells. Electron microscopy analysis showed that more autolysosomes and autophagosomes accumulated in Beclin 1–2KR-expressing cells (Fig. 5c,d). To further assess autophagosome maturation, we performed western blot analyses of p62, which is an autophagic substrate. In response to starvation, the degradation of p62 was accelerated in the Beclin 1–2KR-expressing MCF7 cells (Fig. 5e). However, treatment with bafilomycin A1 blocked p62 degradation in both the Beclin 1–2KR mutant and WT cells with little difference (Supplementary Fig. 5b); however, when we retracted the inhibitor, p62 decreased more rapidly in the 2KR mutant cells (Supplementary Fig. 5c). Since p62 would be degraded after autophagosome maturation and fusion with lysosome, these data indicate that Beclin 1–2KR enhances autophagosome maturation and autophagic degradation rather than autophagy induction. During autophagy maturation, the expressing of Beclin 1 WT had no statistical differences, which suggested that the function of Beclin 1 must be under more precise control, such as phosphorylation and acetylation. In view of that, autophagosomes were also increased in the cells expressing Beclin 1–2KR mutants by the counting of yellow puncta in mCherry-EGFP-LC3 tandem vector assay and in electron microscopy analysis (Fig. 5b,d). To determine whether Beclin 1 acetylation could also affect autophagosome formation, we also used the GFP-LC3 vector in the cells overexpressing Beclin 1 WT or 2KR mutant. Supplementary Fig. 5d showed that there was only a slight increase in the number of GFP-LC3 puncta in 2KR-mutant cells with or without bafilomycin A1 treatment. The difference, however, was not statistically significant. All the experiments above further demonstrate that 2KR mutant of Beclin 1 promoted the maturation of autophagosome rather than the autophagosome formation.

Given that the autophagic pathway converges with the endocytic pathway at the endosome node for lysosome-dependent degradation^22^, we further examined whether the acetylation of Beclin 1 would affect the endocytic processes. When stimulated with epidermal growth factor (EGF), the degradation of EGF receptor (EGFR) was accelerated in MCF7 cells expressing Beclin 1–2KR compared with MCF7 cells expressing wide-type Beclin 1, and as a negative control, EGFR was more stable in the vector cells compared with that in the Beclin 1-overexpressing ones (Fig. 5f). In addition, as an upstream signal, the Beclin 1 AA mutation could also accelerate p62 degradation compared with Beclin 1 WT; similar effect was also observed in cells with 2KR mutation (Supplementary Fig. 6a). We also found that overexpression or knockdown of CK1γ2 could decelerate or accelerate this process, respectively (Supplementary Fig. 6b,c). Overall, these findings indicate that Beclin 1 acetylation enhances the interaction of Beclin 1 with Rubicon, which subsequently inhibits the intracellular trafficking of autophagosome and endocytic cargo to late endosomes/lysosomes for degradation.

### Acetylation of Beclin 1 inhibits tumour growth

**Figure 6:**
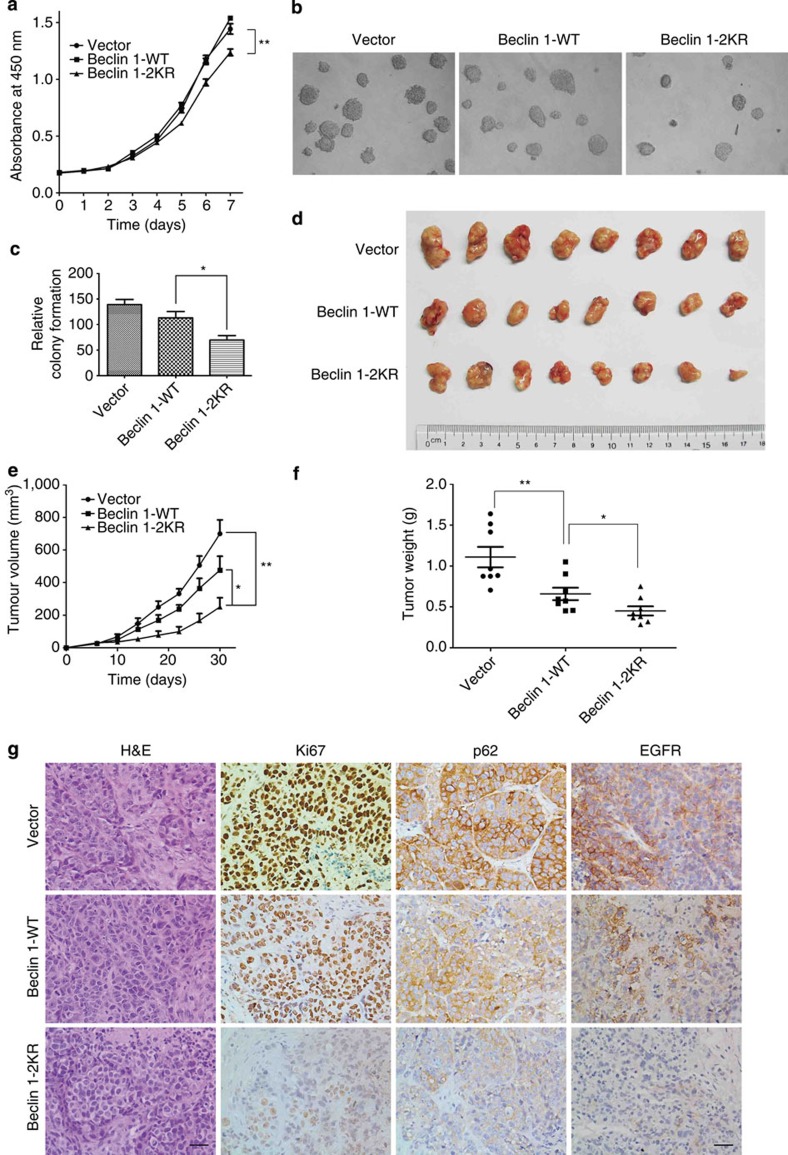
Acetylation of Beclin 1 promotes tumour cell proliferation and tumorigenesis *in vivo*. (**a**) Beclin 1–2KR mutant inhibits cell proliferation. MCF7 cells stably expressing vector control, Beclin 1 WT or 2KR were seeded at the same number in each well. Then made CCK-8 assay every 24 h. Results are mean±s.d. for experiments performed in triplicate, **P<0.01 (Student's *t*-test). (**b**) MCF7 cells stably expressing vector control, Beclin 1 WT or 2KR were used for colony formation assays. Cells (0.5 × 10^4^) were grown in soft agar, and the colonies were monitored after 2 weeks. Representative pictures of the colonies are shown. Results from three independent experiments are presented as means±s.d., *P<0.05 (Student's *t*-test). (**d**) Beclin 1–2KR mutant inhibits xenograft tumour growth. MCF7 cell lines (6 × 10^6^ cells) with vector control, Beclin 1 WT or 2KR were injected subcutaneously into the right flank of nude mice. At 38 days after injection, tumours were extracted and photographed. (**e**) Tumour growth curves in nude mice. Tumour diameters were measured at the indicated time points, and tumour volumes were calculated. Results are mean volume±s.e.m. for 10 mice per group per time point. *P<0.05 (Student's *t*-test). (**f**) Tumour weights from experiment in **c** on autopsy on day 38. *P<0.05 (Student's *t*-test). (**g**) Beclin 1–2KR expression inhibits tumour cell proliferation *in vivo*. Tumour sections from xenografts were prepared for immunohistochemistry analysis. Representative haematoxylin and eosin staining (H&E) and Ki-67, p62, EGFR immunostaining images of indicated MCF7 xenograft tumour genotype. Scale bars, 20 μm.

Given several lines of evidence indicate that decreased Beclin 1 function contributes to tumour initiation^3^,^23^, we next examined the effect of Beclin 1 acetylation on cell proliferation and tumour growth. To this end, we used stable MCF7 cell lines in which vector control, Beclin 1 WT or 2KR was stably expressed (Supplementary Fig. 7). We found that cells expressing Beclin 1–2KR mutant proliferated slower than the cells expressing wild-type Beclin 1 and vector under normal culture conditions (Fig. 6a). To assess anchorage-independent cell growth, we conducted assays of cell growth in soft agar. As shown in Fig. 6b,c, Beclin 1 WT expression formed significantly more and larger colonies than Beclin 1–2KR, but slightly less than the vector group.

To determine whether the acetylation of Beclin 1 also rendered growth advantage to tumour cells *in vivo*, we performed tumour xenograft studies. MCF7 cells with stable expression of vector control, wild-type or 2KR-mutant Beclin 1 were injected into nude mice, and tumour cell growth was monitored over a period of 38 days. Beclin 1–2KR-expressing MCF7 xenografts grew at a slower rate than those derived from MCF7-expressing wild-type Beclin 1 and vector (Fig. 6d–f). As the expression of Beclin 1 2KR mutant showed a more remarkable inhibition on tumour growth than the WT group, we speculated that the tumour suppressor function of Beclin 1 might be achieved through its deacetylation. In addition to the aforementioned features, histopathologic analyses revealed significant differences in MCF7 xenografts expressing Beclin 1–2KR (Fig. 6g). Beclin 1 WT MCF7 xenografts displayed strong Ki-67 staining homogenously in nearly all tumour cells, whereas the majority of tumour cells in Beclin 1–2KR xenografts displayed weak Ki-67 staining. It demonstrated that the tumours derived from Beclin 1 WT-expressing cells were more proliferative than those derived from cells expressing Beclin 1–2KR (Fig. 6g). Moreover, MCF7 Beclin 1–2KR xenografts displayed weaker p62 and EGFR staining than Beclin 1 WT xenografts (Fig. 6g) because of stronger autophagic degradation. Thus, deacetylation of Beclin 1 by the mutation of K430 and K437 sites in MCF7 xenografts results in increased autophagy flux, decreased cellular proliferation and tumour growth. Taken together, these results demonstrate that acetylation of Beclin 1 promotes cell proliferation and tumour growth.

## Discussion

Autophagy contributes to basal cellular and tissue homeostasis and to developmental regulation in higher organisms. Consequently, autophagy must be strictly controlled. The autophagic pathway proceeds through several phases, including initiation, vesicle elongation, autophagosome maturation and autophagosome–lysosome fusion^24^. Beclin 1 can intervene at every major step in autophagic pathways that is mediated by its interacting partners. Therefore, the regulation of the Beclin 1 complex is crucial for autophagy control.

**Figure 7:**
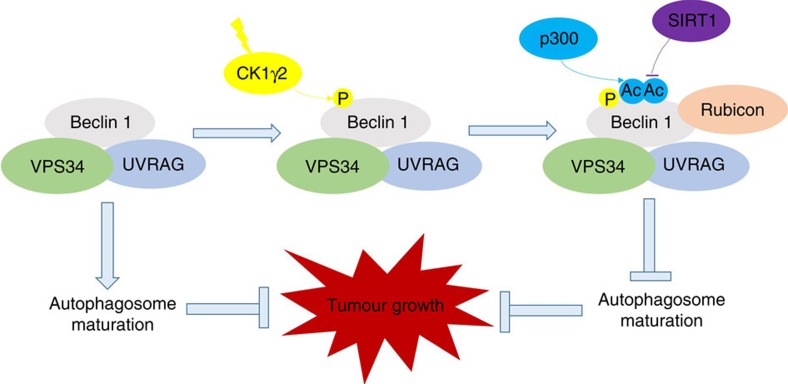
A working model of Beclin 1 acetylation on autophagy and tumour growth.

Phosphorylation is the well-studied modification of Beclin 1. The phosphorylation of Beclin 1 by death-associated protein kinase triggers the dissociation of the Beclin 1–Bcl-2 complex allowing Beclin 1 to activate Vps34 (refs 13, 25). Furthermore, the direct phosphorylation of Beclin 1 (S91/S94) or Vps34 (T163/S165) by AMPK can regulate autophagy by forming different Vps34 complexes^26^. ULK1 can phosphorylate Beclin 1 directly at Ser 14 and activates the Vps34 lipid kinase^27^. Those modifications are mostly associated with the regulation of the Beclin 1 complex for autophagy initiation. More recently, it has been shown that Akt and EGFR can inhibit autophagy by phosphorylating Beclin 1 directly and promoting the formation of the inhibitory Beclin 1 complex^23^,^28^. The phosphorylated site of Beclin 1 by CK1γ2 was close to the acetylation site, and neither Akt- nor EGFR-targeting phosphorylation site of Beclin 1 was located within this domain. To eliminate the effect of AKT- or EGFR-targeting phosphorylation, we further examined the acetylation of Beclin 1 and its interaction with Rubicon by overexpressing Akt or EGFR. As shown in Supplementary Fig. 8a, overexpression of Akt could not increase the acetylation of Beclin 1. Overexpression of EGFR, however, could increase the Beclin 1 acetylation and Beclin 1–Rubicon interaction, which was through CK1γ2 regulation as inhibiting of CK1γ2 could reverse this effect (Supplementary Fig. 8b). Therefore, phosphorylation of Beclin 1 by CK1γ2 was the one necessary for acetylation of Beclin 1 (Fig. 7).

The importance of acetylation in autophagy control has only been realized recently. Studies have shown that treating cells with deacetylase inhibitors can induce autophagy^29^, whereas the p300-mediated acetylation of autophagy-associated proteins appears to inhibit autophagy^15^. It has been reported that the acetylation of Atg5, Atg7, Atg8 and Atg12 by the acetyltransferase p300 results in autophagy inhibition^15^. In addition, the NAD-dependent deacetylase SIRT1 increases basal autophagy by deacetylating Atg5, Atg7 and Atg8 (ref. 30). A recent study reported that autophagy can be regulated by cytosolic acetyl-coenzyme A, and p300 is required for AcCoA-mediated autophagy inhibition^31^.In this study, we found that p300-mediated Beclin 1 acetylation could inhibit autophagosome maturation (Fig. 7). However, the association between protein acetylation and autophagy warrants further investigation.

Acetylation and ubiquitination are both associated with lysine residue, and it has been previously reported that the acetylation residues could also be the ubiquitylation residues in different conditions. For example, the acetylation of Lys437 on Beclin 1 presented in the present study has also been previously identified as an ubiquitylation site^32^. To assess the interplay between these two modifications, we examined the polyubiquitination of Beclin 1 in the presence of TSA/NAM, results of which suggested that the deacetylase inhibitors could not affect the ubiquitination of Beclin 1 (Supplementary Fig. 9).

Casein Kinase 1 is a family of evolutionarily conserved serine/threonine kinases that regulate signal transduction in most eukaryotic cell types. CK1 isoforms are the key regulators of several cellular growth and survival processes, including Wnt and Hedgehog signalling, cell cycle control, DNA repair and apoptosis^33^. Several studies suggest that CK1 plays an important role in oncogenesis, which maybe a result of the combinatorial deregulation of these cellular processes. Here we found that CK1γ2 can phosphorylate Beclin 1 at S409 and that S409 phosphorylation is dependent on the priming phosphorylation of T406. However, the kinase that targets T406 is yet to be determined. CK1γ2-mediated Beclin 1 phosphorylation promotes the subsequent acetylation of Beclin 1 by p300, and acetylation of Beclin 1 promotes cell proliferation and tumour growth. Our present findings suggest that CK1γ2 may implicate in tumour progression via promoting Beclin 1 acetylation and suppression of autophagosome maturation (Fig. 7).

In summary, our current knowledge on Beclin 1 suggests that distinct post-translational modification of Beclin 1, which can occur at different autophagy stages, may lead to the induction, inhibition or fine-tuning of the autophagic response under a variety of conditions. Moreover, many studies indicate that decreased Beclin 1 function contributes to tumour growth. In this study, we identify an acetylation-dependent regulatory mechanism governing Beclin 1 function in autophagy for the first time, and Beclin 1 acetylation can inhibit autophagosome maturation and promote tumour growth. Our research further clarifies the mechanism of Beclin 1-regulating autophagosome maturation, offering new targets and ideas for tumour therapy.

## Methods

### Cell culture

HEK293T, MCF7 and HeLa cells were purchased from American Type Cell Culture (Manassas, VA). HEK293T and MCF7 cells were cultured in high-glucose (25 mM) DMEM medium containing 10% fetal bovine serum (Gibco) and 50 mg ml^−1^ penicillin/streptomycin. HeLa cells were grown in RPMI-1640 medium with 10% fetal bovine serum (Gibco).

### Antibodies and reagents

Anti-Vps34 (1:1,000, no. 4263), Beclin 1 (1:1,000, no. 3738), acetylated lysine (1:500, no. 9441), haemagglutinin (HA)-Tag (1:1,000, no. 3724, Rabbit) and HA-Tag (1:1,000, no. 2367, Mouse) antibodies were purchased from Cell Signaling Technology. Anti-UVRAG (1:1,000, NBP1–18885), Beclin 1 (1:1,000, NBP1–00088) and LC3 (1:5,000, NB100–2220) antibodies were obtained from NOVUS. Anti-Rubicon (1:1,000, ab92388) and p150 (1:1,000, ab128903) antibodies were obtained from Abcam. Anti-SIRT1 (1:1,000, AJ1717a), SIRT2 (1:1,000, AJ1718a) and HDAC1 (1:1,000, AP1101a) antibodies were purchased from Abgent. Anti-p300 (1:500, sc-585), p62/SQSTM1 (1:2,000, sc-28359) and HDAC2 (1:1,000, sc-55542) antibodies were purchased from Santa Cruz Biotechnology. Anti-a-tubulin (1:2,000, T6199), Flag (1:5,000, F3165) and Atg14L (1:1,000, A6358) antibodies were obtained from Sigma. Anti-phospho S409 Beclin 1 antibodies were generated by immunizing rabbits with the corresponding phosphopeptides. Bafilomycin A1, rapamycin, deacetylase inhibitors TSA and NAM, and CK1 inhibitor D4476 were purchased from Sigma.

### Western blotting and immunoprecipitation

Whole-cell extracts were generated by direct lysis with 1 × Cell Lysis Buffer (Cell Signaling Technology, no. 9873) adding 1 mM phenylmethylsulphonyl fluoride (PMSF) immediately before use. Samples were boiled by addition 6 × SDS sample buffer for 10 min at 100 °C and resolved using SDS–PAGE. For immunoprecipitation, cells were lysed by E1A lysis buffer (250 mM NaCl, 50 mM HEPES (pH 7.5), 0.1% NP-40, 5 mM EDTA, protease inhibitor cocktail (Roche)). For acetylation immunoprecipitation, 2 μM TSA and 10 mM NAM were added. Lysates were cleared by centrifugation at 12,000 r.p.m. for 15 min at 4 °C. Immunoprecipitation was carried out either by incubating FLAG beads at 4 °C with lysate overnight or by incubating appropriate antibody with cell lysate for 2–3 h, followed by incubating Protein-A/G beads overnight (Roche). Immunoprecipitates were washed three times with cold lysis buffer and eluted with SDS loading buffer by boiling for 10 min. Uncropped images of immunoblots presented in the main paper are provided in Supplementary Information (Supplementary Fig. 10).

### Plasmids and transfection

The following Addgene plasmids were used: Addgene plasmid 30,489 (pCMVβ-p300-myc), Addgene plasmid 8,941 (pCI flag pCAF), Addgene plasmid 33,338(HTATIP(2OU2)), Addgene plasmid 16,701 (pRc/RSV-mCBP-HA), Addgene plasmid 1,791 (Flag-SIRT1), Addgene plasmid 1,792 (Flag-SIRT1 H363Y), Addgene plasmid 23,705 (pDONR223-CSNK1G2), Addgene plasmid 22,418 (pBABE-puro mCherry-EGFP-LC3B) and Addgene plasmid 22,405 (pBABE-puro GFP-LC3). Some of these plasmids were constructed into another destination vector or added a different epitope tag of choice. pBabe-HA-Beclin 1 was constructed by subcloning pcDNA3-Beclin 1 constructs into pBabe retroviral vector. Site-directed mutagenesis of Flag-Beclin 1 was performed using QuikChange XL (Stratagene). Plasmid transient transfection was performed using Lipofectamine 2,000 according to the manufacturer's instructions (Invitrogen). To generate retroviruses, 293GPG-packaging cells were transfected with empty vector (pBABE-puro) or HA-tagged pBABE-Beclin 1 constructs using Lipofectamine 2,000 (Invitrogen). Medium was changed 24 h after transfection, and the cell supernatant was collected on days 3 and 4. The cleared supernatant was filtered through a 0.45-μm filter, aliquoted and stored at −80 °C. Target cells were infected by retrovirus supernatant in the presence of 8 μg ml^−1^ Polybrene every 12 h for three rounds. After infection, the cells were selected using 1 μg ml^−1^ puromycin for 3 days.

### RNAi

All siRNAs were produced by GenePharma and transfected using Lipofectamine RNAiMAX Transfection Reagent (life Technologies) according to the manufacturer's protocol. Target sequences for siRNAs are as follows:

HDAC1 (5′-CAGCGACUGUUUGAGAACC-3′),

HDAC2 (5′-CAGTCTCACCAATTTCAGAAA-3′),

SIRT1 no. 1 (5′-GCTAAGAATTTCAGGATTA-3′),

SIRT1 no. 2 (5′-ACUUUGCUGUAACCCUGUA-3′),

SIRT2 (5′-GACTCCAAGAAGGCCTACA-3′),

p300 (5′-CAGACAAGTCTTGGCATGGTA-3′),

CK1γ2 no. 1 (5′-GCUAAAGGCUGCAACAAAG-3′),

CK1γ2 no. 2 (5′-CAAUAUGACUACACAUUUG-3′),

### Autophagy analysis

Autophagy was measured by quantitation of GFP-LC3 puncta using fluorescence microscopy. Cells were infected with appropriate amounts of lentivirus carrying GFP-LC3 to express the close-to-endogenous level of GFP-LC3. After treatment, cells were fixed with 4% paraformaldehyde for 20 min and rinsed with PBS twice. Cells were mounted and visualized under a confocal microscope (Olympus FV-1,000). Total number of cells on images was determined by nuclei staining with 4,6-diamidino-2-phenylindole. Autophagosome maturation was assessed by transfecting the mCherry-EGFP-LC3 tandem vector. The percentage of red-only puncta was quantitated.

### Mass spectrometry

HEK293T cells were transfected with Flag-tagged Beclin 1 expression plasmids and cultured for 20 h, then the transfected HEK293T cells were treated with 1 mM TSA and 5 mM NAM for 6 h before harvest. Cells were collected and lysed with E1A lysis buffer plus 2 mM TSA and 10 mM NAM, and the cell extracts were immunoprecipitated with the anti-Flag monoclonal antibody-conjugated M2 agarose beads (Sigma-Aldrich). The Flag peptide-eluted material was resolved by 8% SDS–PAGE. The Beclin 1 bands were excised from the gel and subjected to tryptic digestion and mass spectrometry. Protein and modification identification was performed with the database search, and peptide identifications were validated with Peptide Prophet.

### *In vitro* acetylation and deacetylation assay

The acetylation reaction was performed in 30 μl of reaction mixture containing 20 mM Tris-HCl (pH 8.0), 20% glycerol, 100 mM KCl, 1 mM dithiothreitol (DTT), 0.1 mM EDTA, 10 μM TSA, 5 mM NAM, 1 mM PMSF, 100 μM acetyl-CoA and 1 μg purified p300 protein (Enzo Life Sciences) and 2 μg of GST-tagged Beclin 1 protein. After incubation at 30 °C for 1 h, the reaction was stopped by the addition of 6 μl of 6 × SDS sample buffer. The samples were subjected to SDS–PAGE and analysed using immunoblotting.

293T cells were transfected with vector Flag-Beclin 1 WT, and then treated with TSA (1 μM) and NAM (5 mM) for 6 h before harvest. The deacetylation reaction was performed in 50 μl of reaction mixture containing Tris-HCl (pH 8.8), 5% glycerol, 50 mM NaCl, 4 mM MgCl, 1 mM DTT and 0.3 μg active SIRT1 protein (Sigma) and Flag-Beclin 1 immunoprecipitated by 30 μl ANTI-FLAG M2 Affinity Gel (Sigma). During the incubation at 37 °C for 0.5 h, the buffer was mixed per 5 min and the reaction was stopped by addition of 10 μl of 6 × SDS sample buffer. The samples were subjected to SDS–PAGE and analysed by immunoblotting.

### GST–Beclin 1 fusion protein purification

The bacterial expression constructs (pGEX-4T-2) containing the indicated genes were transformed into BL21-competent cells (Agilent Technologies). Cells were induced to protein overexpression under 0.1–0.5 mM isopropyl-b-D-thiogalactoside at 37 °C. Cells were resuspended in PBS containing 0.5% Triton X-100, 5 mM β-mercaptoethanol, 2 mM EDTA and 1 mM PMSF, followed by ultrasonication. The proteins were purified by a single step using glutathione bead according to the manufacturer's protocol (GE Health Science).

### EGFR degradation assay

Cells cultured in six-well plates were grown to ∼80% confluency, washed with PBS and serum-starved for 24 h. EGFR endocytosis was stimulated by addition of EGF (200 ng ml^−1^, Life Technologies). At each time point after EGF stimulation, the cells were lysed and equal amounts of the lysate were analysed using SDS–PAGE and immunoblotted with anti-EGFR antibody (1:1,000; Cell Signaling Technology).

### Anchorage-independent growth assay

For each well of a six-well plate, cells (5 × 10^3^) were mixed with a medium containing 0.35% agar and were spread on top of a bottom agar layer (0.6% agar in growth medium). Fresh layer of the medium containing 0.35% agar was added weekly on top of the previous layer. Colonies were monitored after 2 weeks.

### Cell proliferation analysis

Briefly, a total of 500 MCF7 stable cells were seeded in triplicate in each well of a 96-well plate, and the cell numbers were counted every day by CCK-8 over a week.

### Tumour xenograft analysis

Nude mice (nu/nu, 6-week-old females) were injected subcutaneously in the right flank region with 6 × 10^6^ MCF7 stable cells. Tumour volume was measured every 5 days using the formula (tumour volume=*π*/6 (*L* × *W*^2^)) for 38 days. Mice were killed on day 38, and the tumours were dissected and analysed. All animal experiments complied with ethical regulations and were approved by the Subcommittee on Research Animal Care of Sun Yat-sen University.

### Immunohistochemistry

Xenograft tumours were fixed in 4% paraformaldehyde (PFA), embedded in paraffin, sectioned and stained with haematoxylin and eosin. Immunohistochemical staining of paraffin-embedded tumour tissues was performed using p62 (Santa Cruz, 1:100 dilution) and Ki-67 (Abcam, 1:100 dilution) primary antibodies and the ABC Elite immunoperoxidase kit according to the manufacturers' instructions.

### Statistical analyses

Student's *t*-test was used to compare the differences between two groups. *P<0.05 was considered statistically significant and **P<0.01 as highly significant.

## Additional information

**How to cite this article:** Sun, T. *et al.* Acetylation of Beclin 1 inhibits autophagosome maturation and promotes tumour growth. *Nat. Commun.* 6:7215 doi: 10.1038/ncomms8215 (2015).

## Supplementary Material

Supplementary Information Supplementary Figures 1-10 and Supplementary Table 1.

## References

[b1] Mizushima N. & Komatsu M. Autophagy: renovation of cells and tissues. Cell 147, 728–741 (2011). 22078875 10.1016/j.cell.2011.10.026

[b2] Levine B. & Klionsky D. J. Development by self-digestion: molecular mechanisms and biological functions of autophagy. Dev. Cell 6, 463–477 (2004). 15068787 10.1016/s1534-5807(04)00099-1

[b3] Levine B. & Kroemer G. Autophagy in the pathogenesis of disease. Cell 132, 27–42 (2008). 18191218 10.1016/j.cell.2007.12.018 PMC2696814

[b4] Mizushima N., Levine B., Cuervo A. M. & Klionsky D. J. Autophagy fights disease through cellular self-digestion. Nature 451, 1069–1075 (2008). 18305538 10.1038/nature06639 PMC2670399

[b5] Shintani T. & Klionsky D. J. Autophagy in health and disease: a double-edged sword. Science 306, 990–995 (2004). 15528435 10.1126/science.1099993 PMC1705980

[b6] Zhong Y. *et al.* Distinct regulation of autophagic activity by Atg14L and Rubicon associated with Beclin 1-phosphatidylinositol-3-kinase complex. Nat. Cell Biol. 11, 468–476 (2009). 19270693 10.1038/ncb1854 PMC2664389

[b7] Backer J. M. The regulation and function of Class III PI3Ks: novel roles for Vps34. Biochem. J. 410, 1–17 (2008). 18215151 10.1042/BJ20071427

[b8] Takahashi Y. *et al.* Bif-1 interacts with Beclin 1 through UVRAG and regulates autophagy and tumorigenesis. Nat. Cell Biol. 9, 1142–1151 (2007). 17891140 10.1038/ncb1634 PMC2254521

[b9] Sun Q. *et al.* Identification of Barkor as a mammalian autophagy-specific factor for Beclin 1 and class III phosphatidylinositol 3-kinase. Proc. Natl Acad. Sci. USA 105, 19211–19216 (2008). 19050071 10.1073/pnas.0810452105 PMC2592986

[b10] Liang C. *et al.* Autophagic and tumour suppressor activity of a novel Beclin 1-binding protein UVRAG. Nat. Cell Biol. 8, 688–699 (2006). 16799551 10.1038/ncb1426

[b11] Di Bartolomeo S. *et al.* The dynamic interaction of AMBRA1 with the dynein motor complex regulates mammalian autophagy. J. Cell Biol. 191, 155–168 (2010). 20921139 10.1083/jcb.201002100 PMC2953445

[b12] Pattingre S. *et al.* Bcl-2 antiapoptotic proteins inhibit Beclin 1-dependent autophagy. Cell 122, 927–939 (2005). 16179260 10.1016/j.cell.2005.07.002

[b13] Zalckvar E. *et al.* DAP-kinase-mediated phosphorylation on the BH3 domain of beclin 1 promotes dissociation of beclin 1 from Bcl-XL and induction of autophagy. EMBO Rep. 10, 285–292 (2009). 19180116 10.1038/embor.2008.246 PMC2658558

[b14] Matsunaga K. *et al.* Two Beclin 1-binding proteins, Atg14L and Rubicon, reciprocally regulate autophagy at different stages. Nat. Cell Biol. 11, 385–396 (2009). 19270696 10.1038/ncb1846

[b15] Lee I. H. & Finkel T. Regulation of autophagy by the p300 acetyltransferase. J. Biol. Chem. 284, 6322–6328 (2009). 19124466 10.1074/jbc.M807135200 PMC5405322

[b16] Zhao Y. *et al.* Cytosolic FoxO1 is essential for the induction of autophagy and tumour suppressor activity. Nat. Cell Biol. 12, 665–675 (2010). 20543840 10.1038/ncb2069

[b17] Zhao S. *et al.* Regulation of cellular metabolism by protein lysine acetylation. Science 327, 1000–1004 (2010). 20167786 10.1126/science.1179689 PMC3232675

[b18] Fujimoto H. *et al.* A possible overestimation of the effect of acetylation on lysine residues in KQ mutant analysis. J. Comput. Chem. 33, 239–246 (2012). 22072565 10.1002/jcc.21956

[b19] Obenauer J. C., Cantley L. C. & Yaffe M. B. Scansite 2.0: Proteome-wide prediction of cell signaling interactions using short sequence motifs. Nucleic Acids Res. 31, 3635–3641 (2003). 12824383 10.1093/nar/gkg584 PMC168990

[b20] Flotow H. *et al.* Phosphate groups as substrate determinants for casein kinase I action. J. Biol. Chem. 265, 14264–14269 (1990). 2117608

[b21] Klionsky D. J. *et al.* Guidelines for the use and interpretation of assays for monitoring autophagy. Autophagy 8, 445–544 (2012). 22966490 10.4161/auto.19496 PMC3404883

[b22] Liou W., Geuze H. J., Geelen M. J. & Slot J. W. The autophagic and endocytic pathways converge at the nascent autophagic vacuoles. J. Cell Biol. 136, 61–70 (1997). 9008703 10.1083/jcb.136.1.61 PMC2132457

[b23] Wang R. C. *et al.* Akt-mediated regulation of autophagy and tumorigenesis through Beclin 1 phosphorylation. Science 338, 956–959 (2012). 23112296 10.1126/science.1225967 PMC3507442

[b24] Choi A. M., Ryter S. W. & Levine B. Autophagy in human health and disease. N. Engl. J. Med. 368, 1845–1846 (2013). 23656658 10.1056/NEJMc1303158

[b25] Wei Y., Pattingre S., Sinha S., Bassik M. & Levine B. JNK1-mediated phosphorylation of Bcl-2 regulates starvation-induced autophagy. Mol. Cell 30, 678–688 (2008). 18570871 10.1016/j.molcel.2008.06.001 PMC2478643

[b26] Kim J. *et al.* Differential regulation of distinct Vps34 complexes by AMPK in nutrient stress and autophagy. Cell 152, 290–303 (2013). 23332761 10.1016/j.cell.2012.12.016 PMC3587159

[b27] Russell R. C. *et al.* ULK1 induces autophagy by phosphorylating Beclin-1 and activating VPS34 lipid kinase. Nat. Cell Biol. 15, 741–750 (2013). 23685627 10.1038/ncb2757 PMC3885611

[b28] Wei Y. *et al.* EGFR-mediated Beclin 1 phosphorylation in autophagy suppression, tumor progression, and tumor chemoresistance. Cell 154, 1269–1284 (2013). 24034250 10.1016/j.cell.2013.08.015 PMC3917713

[b29] Robert T. *et al.* HDACs link the DNA damage response, processing of double-strand breaks and autophagy. Nature 471, 74–79 (2011). 21368826 10.1038/nature09803 PMC3935290

[b30] Lee I. H. *et al.* A role for the NAD-dependent deacetylase Sirt1 in the regulation of autophagy. Proc. Natl Acad. Sci. USA 105, 3374–3379 (2008). 18296641 10.1073/pnas.0712145105 PMC2265142

[b31] Marino G. *et al.* Regulation of autophagy by cytosolic acetyl-coenzyme a. Mol. Cell 53, 710–725 (2014). 24560926 10.1016/j.molcel.2014.01.016

[b32] Xia P. *et al.* WASH inhibits autophagy through suppression of Beclin 1 ubiquitination. EMBO J. 32, 2685–2696 (2013). 23974797 10.1038/emboj.2013.189 PMC3801434

[b33] Eide E. J. & Virshup D. M. Casein kinase I: another cog in the circadian clockworks. Chronobiol. Int. 18, 389–398 (2001). 11475410 10.1081/cbi-100103963

