# Supplementary material for: Acetylation of Beclin 1 inhibits autophagosome maturation and promotes tumour growth
Source: Nat Commun. 2015 May 26;6:7215. doi: 10.1038/ncomms8215 (PMC4455096; doi:10.1038/ncomms8215)
Supplement: Supplementary Information — Supplementary Figures 1-10 and Supplementary Table 1. [file ncomms8215-s1.pdf]

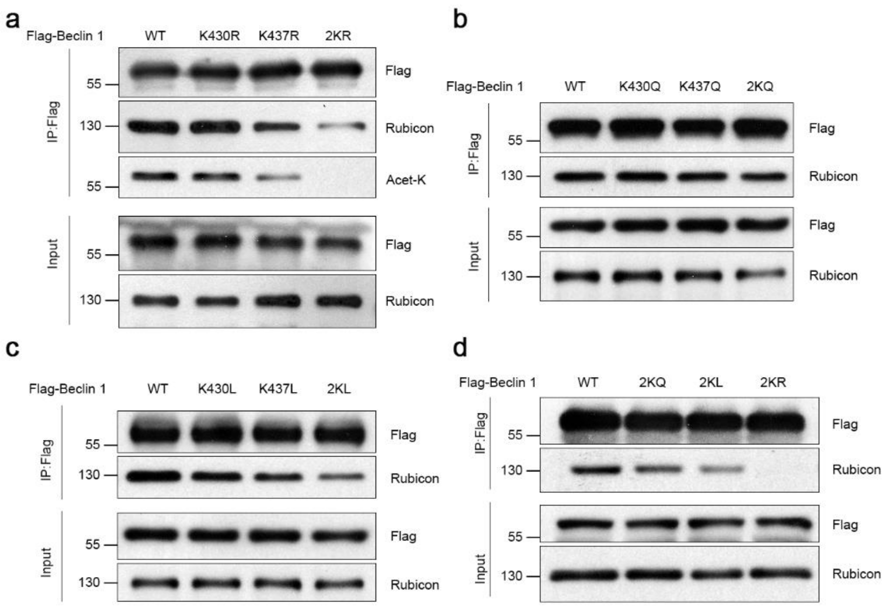

Supplementary Figure 1 The effect of Beclin 1 mutations on Beclin 1 /Rubicon interaction

(a) Mutations of K430R and K437R decrease Beclin 1 acetylation and the binding of Beclin 1 and Rubicon. Immunoprecipitation of indicated proteins with Beclin 1 in MCF-7 cells transfected with indicated Beclin 1 constructs. The Beclin 1/Rubicon interaction and the acetylation were detected by Western blot as indicated. (b) Mutations of Lys430 and Lys437 to glutamine had no obvious effect on the Beclin 1/Rubicon interaction. The interaction between Rubicon and ectopically expressed WT, K430Q, K437Q and 2KQ Beclin 1 was analyzed. (c) Mutations of Lys430 and Lys437 to leucine slightly decreased the interaction between Beclin 1 and Rubicon. The interaction between Rubicon and ectopically expressed WT, K430L, K437L and 2KL Beclin 1 was analyzed. (d) The comparison among three kinds of mutations at K430 and K437. Immunoprecipitation of indicated proteins with Beclin 1 in HEK293T cells transfected with indicated Beclin 1 constructs.

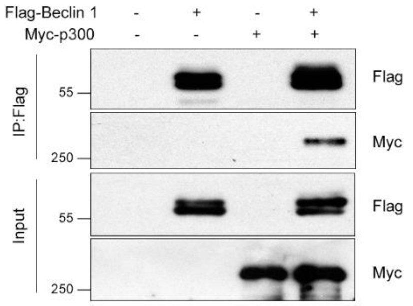

Supplementary Figure 2 Association of Beclin 1 with p300

Flag-tagged Beclin 1 and Myc-tagged p300 were transfected into HEK293T cells individually or together. The interaction between Beclin 1 and p300 was determined by IP and Western blot.

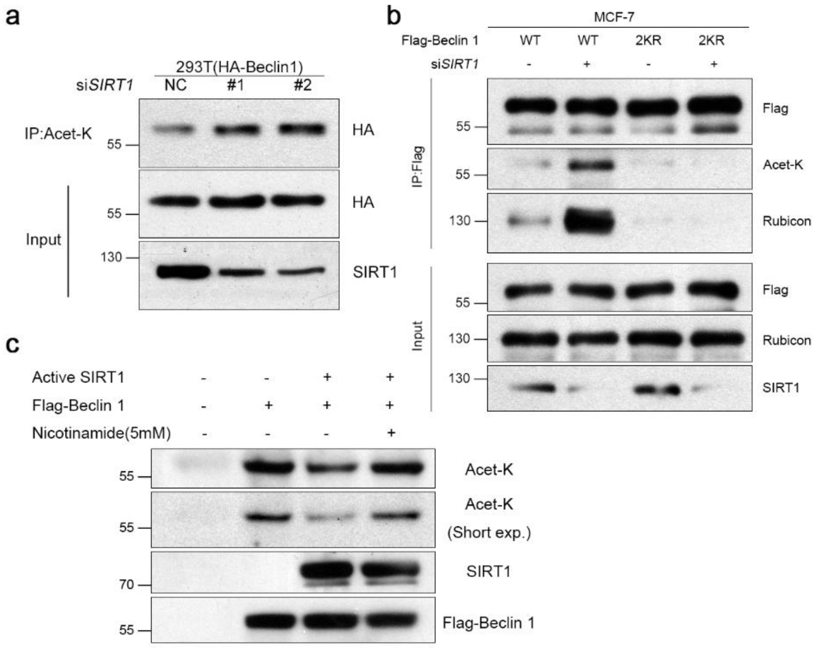

Supplementary Figure 3 SIRT1 is the deacetylase of Beclin 1

(a) Knockdown of SIRT1 increased Beclin 1 acetylation. HEK293T cells stably expressing HA-Beclin 1 were transfected with siRNA targeting SIRT1 or negative control. Beclin 1 acetylation was measured by immunoprecipitation using an anti-acetylated lysine antibody. (b) Beclin 1 2KR mutant counteracted si*SIRT1* acetylation of Beclin 1 and diminished the Beclin 1/Rubicon interaction. Flag-tagged Beclin 1(WT, 2KR) was cotransfected with si*SIRT1* or negative control into MCF-7 cells. (c) Beclin 1 is deacetylated by SIRT1 *in vitro*. Active human SIRT1 protein was incubated with immunoprecipitated Flag-Beclin 1 with or without NAM at 37 ℃ for 0.5 h. The samples were analyzed by immunoblotting.

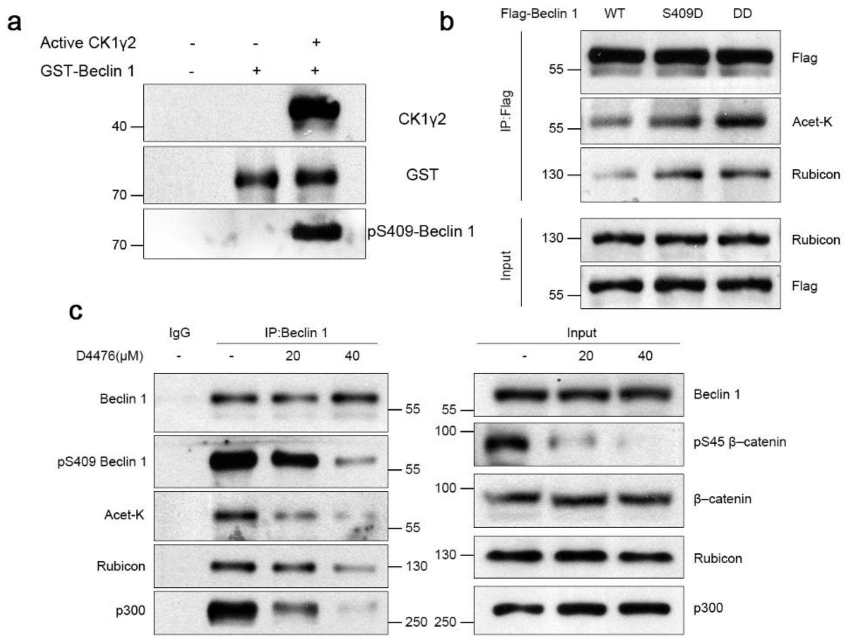

Supplementary Figure 4 CK1-mediated phosphorylation of Beclin 1 is essential for Beclin 1 acetylation

(a) Beclin 1 was phosphorylated by CK1γ2 *in vitro*. Active recombinant human CK1γ2 was incubated with bacterially expressed GST-Beclin 1 in the presence of 400μM ATP for 30min at 30 ℃. The phosphorylation was measured by using the pS409-Beclin 1 antibody. (b) Phosphomimetic mutant of Beclin 1 facilitated acetylation and Beclin 1/Rubicon interacting. HEK293T cells were transfected with indicated Beclin 1constructs. (c) The detection of endogenous Beclin 1 phosphorylation and acetylation. HEK293T cells were treated with D4476 for 4h. Immunoprecipitated Beclin 1 phosphorylation, acetylation and the interaction between Beclin 1 and indicated protein was determined by Westernblot.

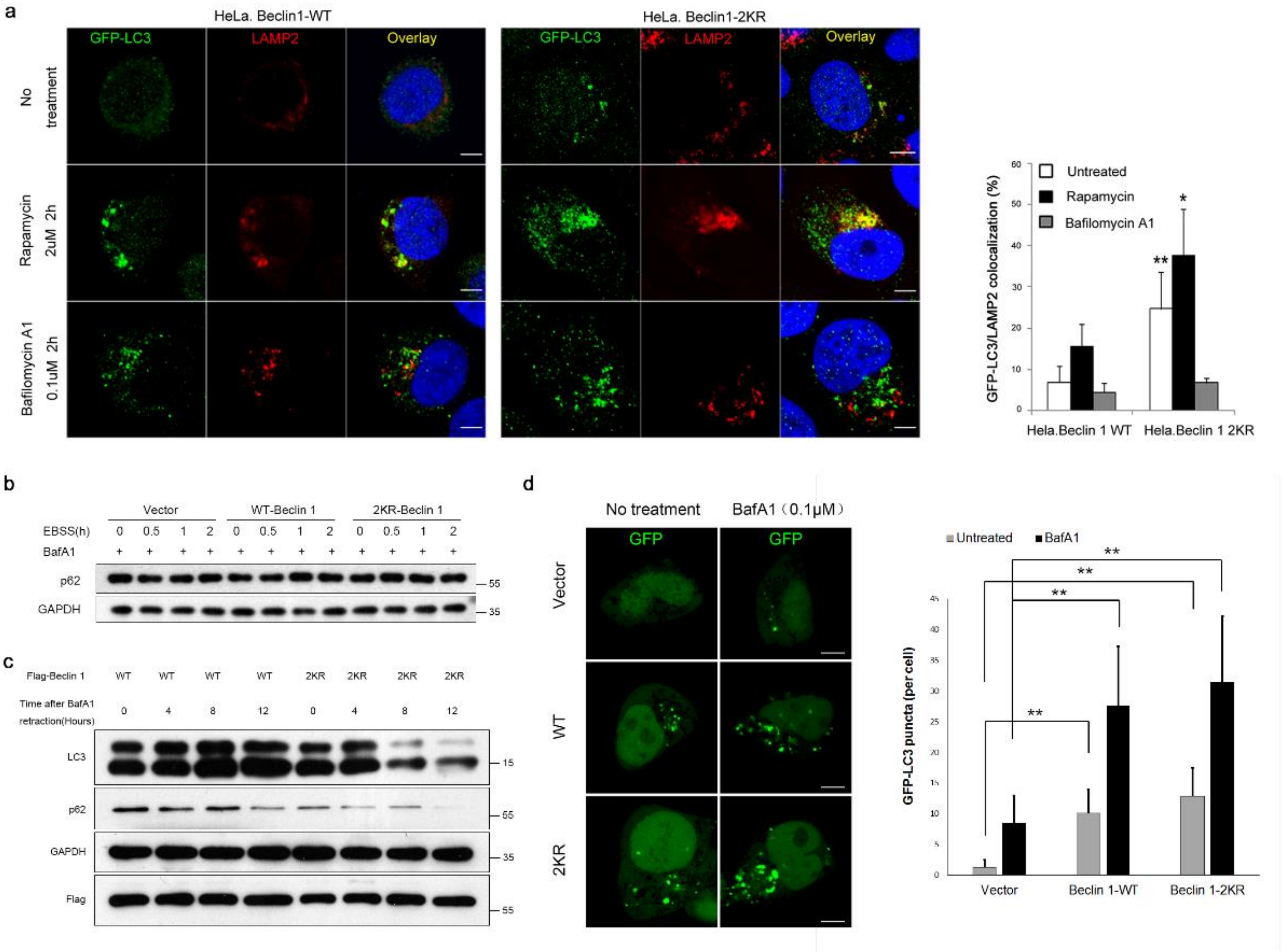

Supplementary Figure 5 The effect of Beclin 1 2KR mutant on Autophagy

(a) Beclin 1 2KR mutant enhances the maturation of autophagosomes. Hela cells stably expressing Beclin 1(WT or 2KR) were transfected with GFP–LC3 and treated with rapamycin (2 μM) in the absence or presence of bafilomycin A1 (0.1 μM) and stained for LAMP2. Scale bars, 5 μm. Bars of quantitation are mean ± SD of 50 cells; 5 independent experiments, **p<0.01; *p<0.05. (b) Treatment with bafilomycin A1 could block p62 degradation in the Beclin 1-2KR-expressing cells and WT ones without distinction. MCF7 cells stably expressing Beclin 1(WT or 2KR) or control were treated with EBSS for the indicated time in the presence of bafilomycin A1 (0.1 μM, 2h). (c) p62 decreased more rapidly in 2KR mutant cells when bafilomycin A1 was revoked. MCF7 cells stably expressing Beclin 1(WT or 2KR) were pretreated with bafilomycin A1 (0.1 μM) for 4h, and revoked the inhibitor for the indicated time. (d) Beclin 1 2KR mutant may not involve in the early stage of autophagy. 293T cells stably expressing vector, Beclin 1 WT or 2KR were transfected with GFP–LC3 and treated with bafilomycin A1 (0.1 μM). Scale bars, 5 μm. Bars of quantitation are mean ± SD of 50 cells; 5 independent experiments, **p<0.01.

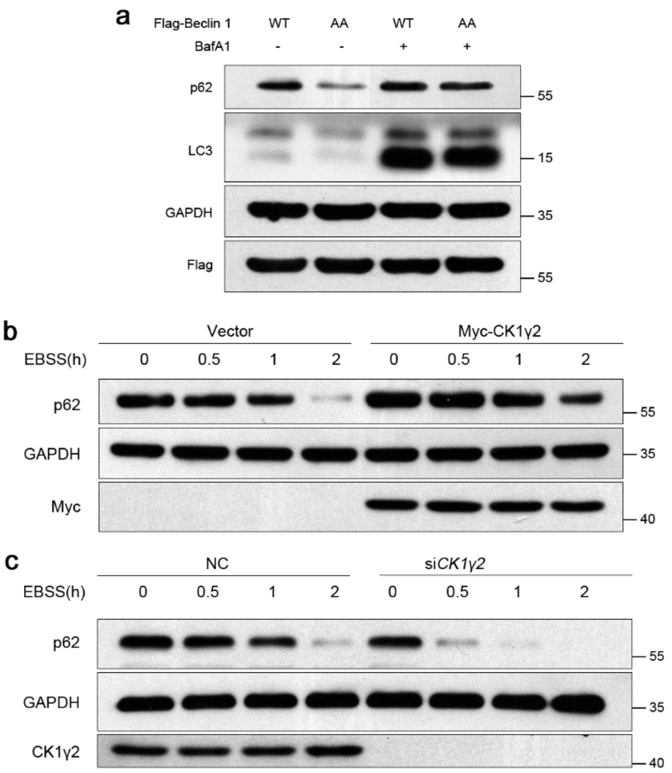

Supplementary Figure 6 The effect of CK1γ2 on autophagosome maturation

(a) AA mutant of Beclin 1 accelerated the degradation of p62. HEK293T cell was transfected with Beclin 1(WT or AA) in the presence or absence of bafilomycin A1 (0.1 μM, 4h). (b) Overexpression of CK1γ2 decelerated the degradation of p62. MCF7 cells were transfected with Myc-CK1γ2 or vector, and treated with EBSS for the indicated times. (c) CK1γ2 knockdown accelerated the degradation of p62. MCF7 cells were transfected with si*CK1γ2* or negative control, and treated with EBSS for the indicated times.

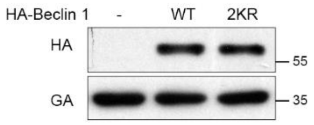

Supplementary Figure 7 The expressing of Beclin 1(WT or 2KR) in MCF7 stable transfecting cells used in Figure 6

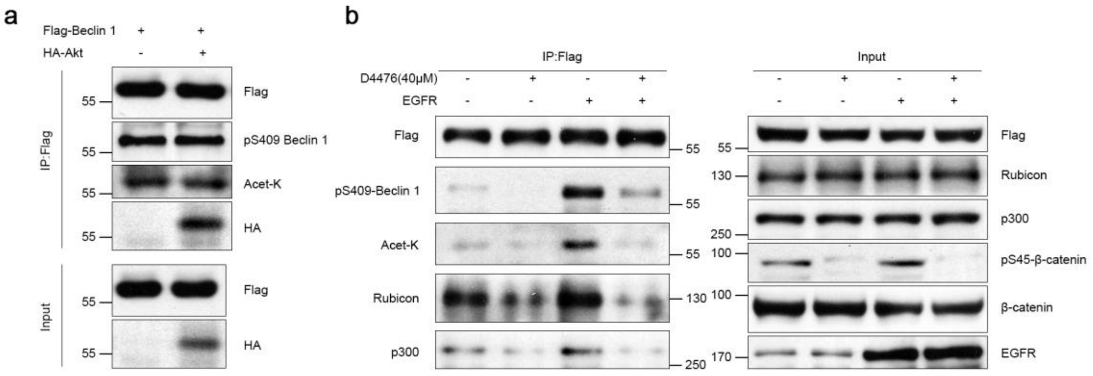

Supplementary Figure 8 Effect of AKT- or EGFR-targeting phosphorylation on Beclin 1 acetylation

(a) Overexpression of Akt could not increase the acetylation of Beclin 1 and Beclin 1/Rubicon interaction. Flag-tagged Beclin 1 was cotransfected with HA-AKT or vector control into HEK293T cells. (b) Overexpression of EGFR, could increase the Beclin 1 acetylation and Beclin 1/Rubicon interaction, and inhibiting of CK1γ2 could reverse this effect. Flag-tagged Beclin 1 was cotansfected with EGFR or control with or without D4476 as indicated.

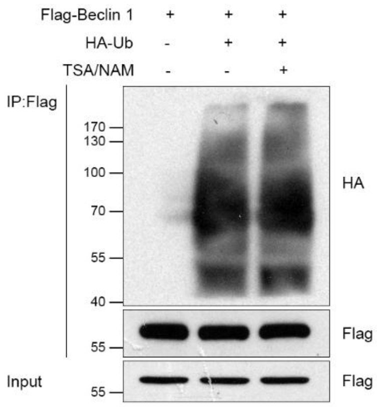

Supplementary Figure 9 The interplay between Beclin 1 polyubiquitination and acetylation

Polyubiquitination of Flag-Beclin 1 immunoprecipitated from HEK293T cells treated with or without TSA (1 μM) and NAM (5mM) for 6 hr was detected.

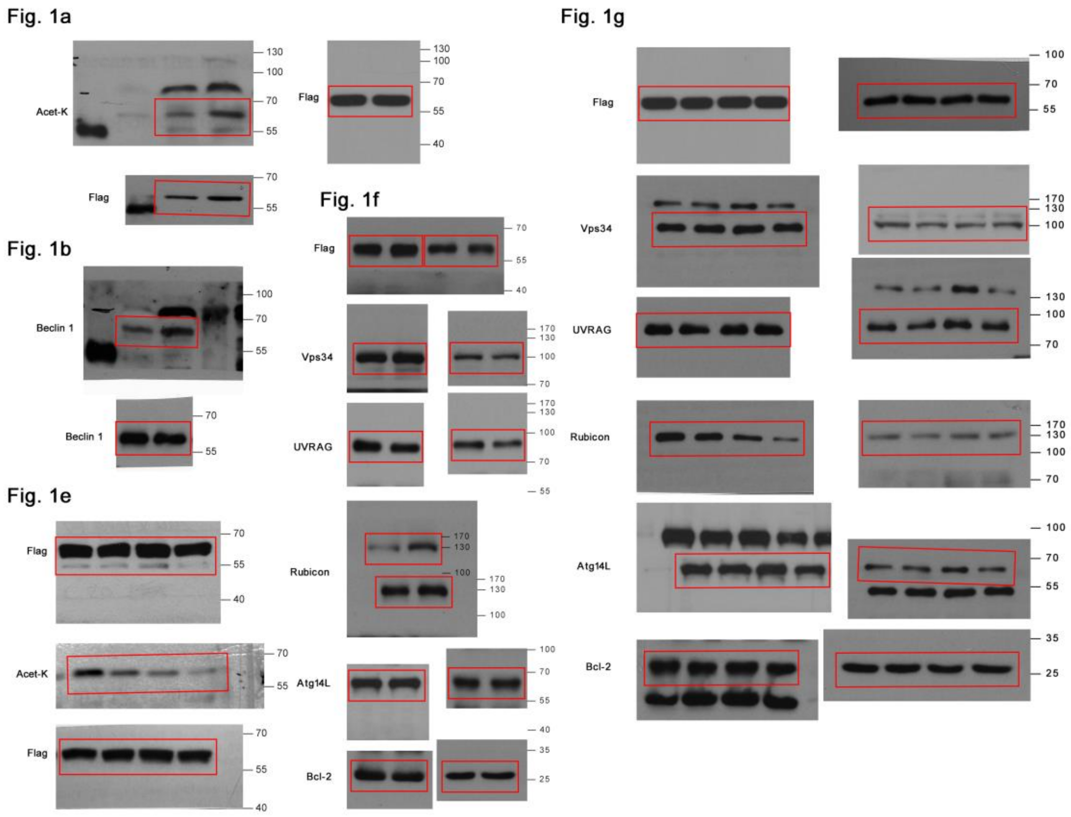

Supplementary Figure 10 Full scans of uncropped blots presented in the main paper

Red boxes indicate the cropped regions. Molecular weight markers are indicated in kDa.

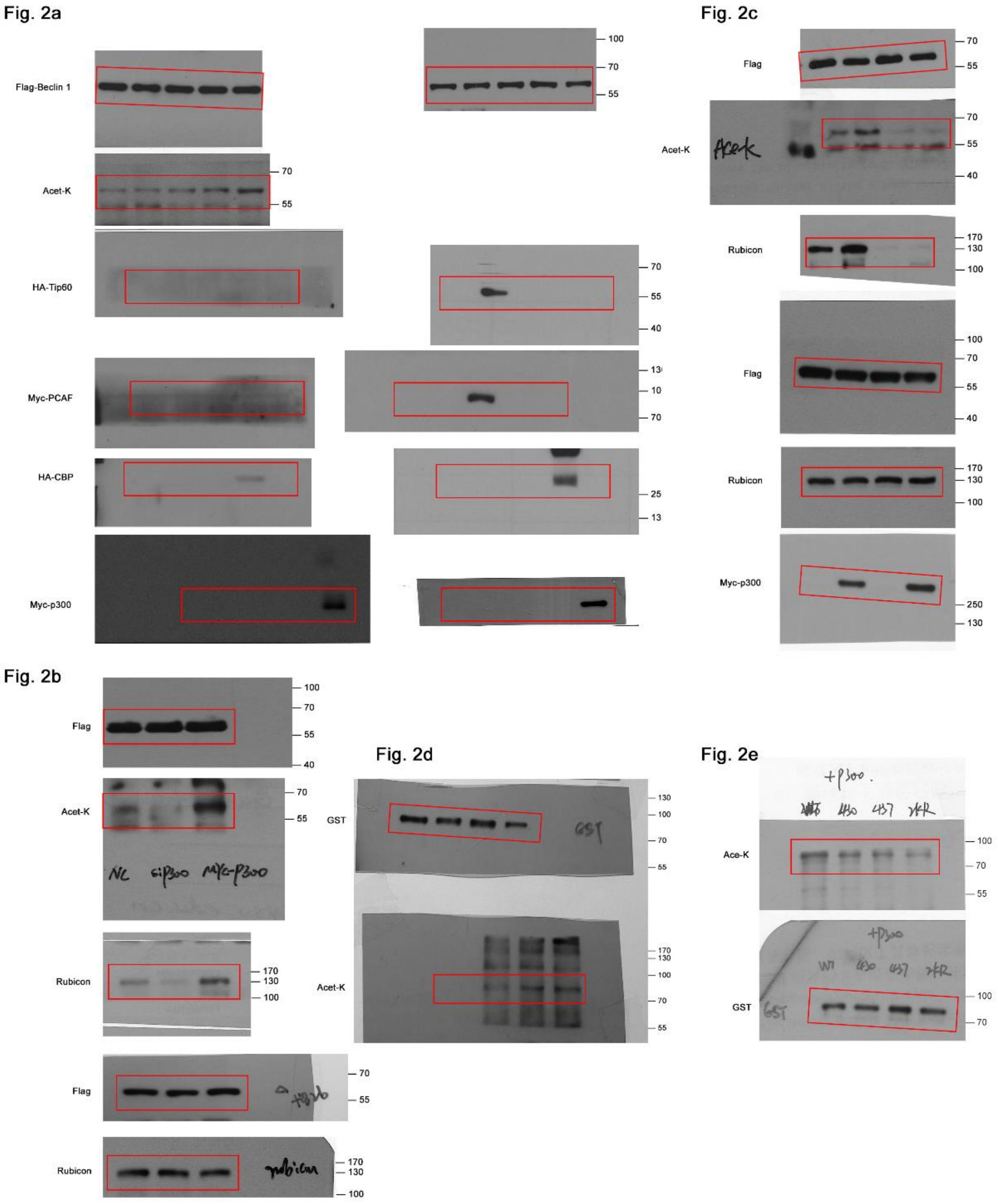

Supplementary Figure 10. continued

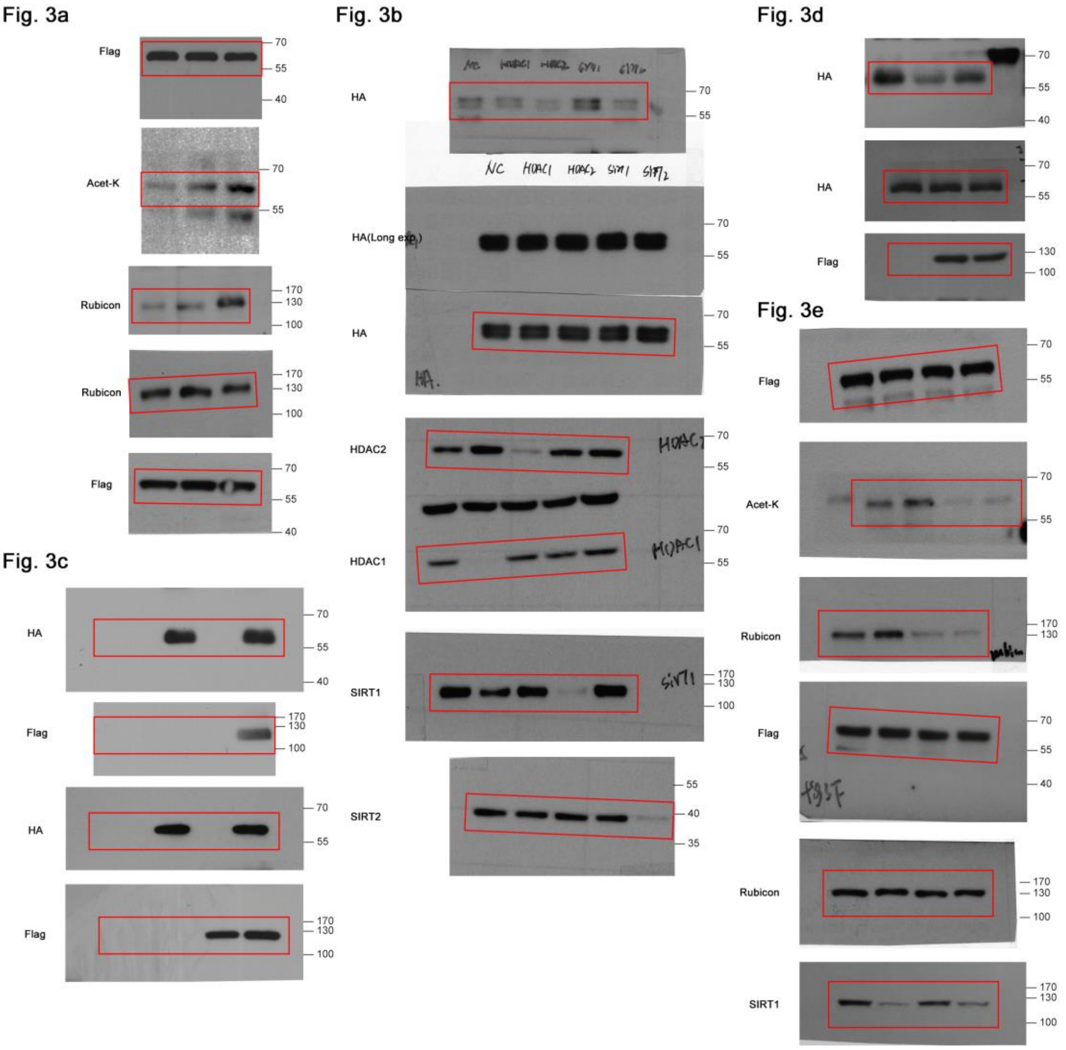

Supplementary Figure 10. continued

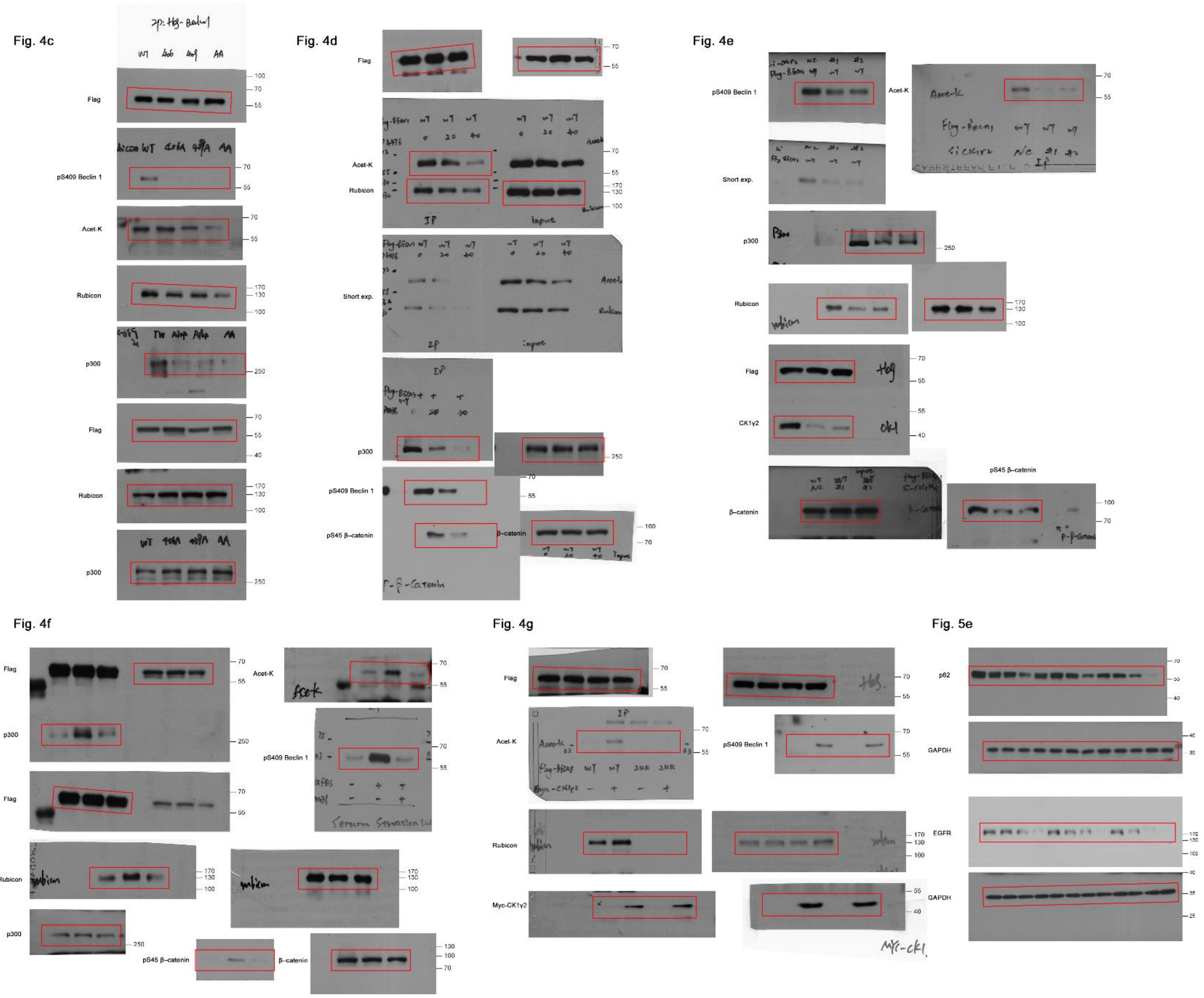

Supplementary Figure 10. continued

Gel Idx/Pos	320/M22	Instr./Gel Origin	AK208/szh 20121129	Process 5tatus	Analysis Succeeded
Plate [#] Name	[1] szh 20121129	Instrument 5ample Name		5pectra	8

Rank	Protein Name	Accession No.	Protein MW	Protein PI	Protein Score	Protein Score C. I. %	Total Ion Score	Total Ion C. I. %
1	Beclin-1 OS=Homo sapiens GN=BECN1 PE=1 SV=2	splQ14457lBECN1 HUMAN	52376.6	4.83	173	100	122	100

Peptide Information

Calc. Mass	Obsrv. Mass	± da	± ppm	Start Seq.	End Seq.	Sequence	Ion Score	C. I. %	Modification	Rank	Result Type
855.4182	855.4279	0.0097	11	390	395	FCLPYR	6	0	Carbamidomethyl (C)[2]		Mascot
855.4182	855.4279	0.0097	11	390	395	FCLPYR			Carbamidomethyl (C)[2]		Mascot
856.5151	856.491	-0.0241	-28	81	87	RFIPPAR					Mascot
958.4628	958.4741	0.0113	12	232	238	EYSEFKR					Mascot
1264.6355	1264.6493	0.0138	11	348	358	ELPLYCSGGLR	37	65.077	Carbamidomethyl (C)[6]		Mascot
1264.6355	1264.6493	0.0138	11	348	358	ELPLYCSGGLR			Carbamidomethyl (C)[6]		Mascot
1479.7625	1479.8011	0.0386	26	346	358	SKELPLYCSGGLR			Carbamidomethyl (C)[8]		Mascot
1635.7261	1635.7886	0.0625	38	401	416	GKIEDTGGSGGSYSIK			Phospho (STY)[6]		Mascot
1715.6925	1715.8184	0.1259	73	401	416	GKIEDTGGSGGSYSIK			Phospho(STY)[9] , Phospho(STY)[6]		Mascot
1715.6925	1715.8184	0.1259	73	401	416	GKIEDTGGSGGSYSIK			Phospho(STY)[9] , Phospho(STY)[6]		Mascot
1758.7898	1758.8071	0.0173	10	6	20	TSNNSTMQVSFVCQR			Carbamidomethyl (C)[13]		Mascot
1758.7898	1758.8071	0.0173	10	6	20	TSNNSTMQVSFVCQR			Carbamidomethyl (C)[13]		Mascot
2062.9788	2062.9922	0.0134	6	215	231	VQAEAERLDQEEAQYQR	25	0			Mascot
2062.9788	2062.9922	0.0134	6	215	231	VQAEAERLDQEEAQYQR					Mascot
2075.0073	2075.0173	0.01	5	239	255	QQLELDDELKSVENQMR	62	99.883			Mascot
2075.0073	2075.0173	0.01	5	239	255	QQLELDDELKSVENQMR					Mascot

Supplementary Table 1 Identification of Beclin 1 T406 and S409 phosphorylation by mass spectrometry analysis

Flag-tagged Beclin 1 was transfected into HEK293T cells. At 24 hr post transfection, HEK293T cells were serum starved for another 24 h, then serum was restored for 2h. Beclin 1 was purified by immunoprecipitation with an anti-Flag antibody and then analyzed by mass spectrometry.
